# Length of Stay After Childbirth in 92 Countries and Associated Factors in 30 Low- and Middle-Income Countries: Compilation of Reported Data and a Cross-sectional Analysis from Nationally Representative Surveys

**DOI:** 10.1371/journal.pmed.1001972

**Published:** 2016-03-08

**Authors:** Oona M. R. Campbell, Luca Cegolon, David Macleod, Lenka Benova

**Affiliations:** Faculty of Epidemiology and Population Health, London School of Hygiene & Tropical Medicine, London, United Kingdom; University of Manchester, UNITED KINGDOM

## Abstract

**Background:**

Following childbirth, women need to stay sufficiently long in health facilities to receive adequate care. Little is known about length of stay following childbirth in low- and middle-income countries or its determinants.

**Methods and Findings:**

We described length of stay after facility delivery in 92 countries. We then created a conceptual framework of the main drivers of length of stay, and explored factors associated with length of stay in 30 countries using multivariable linear regression. Finally, we used multivariable logistic regression to examine the factors associated with stays that were “too short” (<24 h for vaginal deliveries and <72 h for cesarean-section deliveries).

Across countries, the mean length of stay ranged from 1.3 to 6.6 d: 0.5 to 6.2 d for singleton vaginal deliveries and 2.5 to 9.3 d for cesarean-section deliveries. The percentage of women staying too short ranged from 0.2% to 83% for vaginal deliveries and from 1% to 75% for cesarean-section deliveries.

Our conceptual framework identified three broad categories of factors that influenced length of stay: need-related determinants that required an indicated extension of stay, and health-system and woman/family dimensions that were drivers of inappropriately short or long stays. The factors identified as independently important in our regression analyses included cesarean-section delivery, birthweight, multiple birth, and infant survival status. Older women and women whose infants were delivered by doctors had extended lengths of stay, as did poorer women. Reliance on factors captured in secondary data that were self-reported by women up to 5 y after a live birth was the main limitation.

**Conclusions:**

Length of stay after childbirth is very variable between countries. Substantial proportions of women stay too short to receive adequate postnatal care. We need to ensure that facilities have skilled birth attendants and effective elements of care, but also that women stay long enough to benefit from these. The challenge is to commit to achieving adequate lengths of stay in low- and middle-income countries, while ensuring any additional time is used to provide high-quality and respectful care.

## Abstract 2

Oona Campbell and colleagues frame a prediction model for post-partum length of stay and identify factors associated with length of stay in LMIC.

## Editors' Summary

**Background:**

The general recommendation to women, especially to women in resource-poor settings (where more than 95% of all maternal and newborn deaths occur), is to give birth in a health facility. How long women and infants should stay after birth, i.e., the appropriate time to discharge, depends on many factors, but it is known that during the first 24 hours after birth mothers and children are at the highest risk of complications and death. During the postpartum stay, the objectives are to monitor maternal and newborn health, and to provide education on breastfeeding and signs for health problems in mothers and infants. WHO recommends that, in resource-poor settings, mothers and newborns stay for a minimum of 24 hours after vaginal birth.

**Why Was This Study Done?:**

Considerable effort has gone into getting women to give birth in health facilities, and some countries have even made it illegal to give birth at home. However, some reports have suggested that women and infants often spend less than the recommended minimum time after birth in the facility. Actual data on duration of postpartum stays are limited, especially for low- and middle-income countries. This study was done to provide an overview of the reality in all countries for which current data exist, and to explore factors that influence the postpartum length of stay.

**What Did the Researchers Do and Find?:**

The researchers searched databases and recent health surveys covering multiple countries for data on length of stay after facility births. Data for 40 high- and middle-income countries were available in databases compiled by the Organisation for Economic Co-operation and Development (OECD). The Demographic and Health Surveys (DHS) did not report on length of stay, but the researchers used its electronically available datasets to conduct their own analyses for 30 low- and middle-income countries. In addition, two survey programs, the Multiple Indicator Cluster Surveys (MICS) and the US Centers for Disease Control and Prevention Reproductive Health Survey (CDC-RHS), reported relevant data on an additional 21 countries and one country, respectively.

The 92 countries for which they were able to obtain data included 45 middle-income and ten low-income countries (individual sources provided only some of the data that the researchers were interested in). Across countries, the mean length of stay ranged from 0.5 days (in Egypt) to 6.2 days (in Ukraine) for singleton vaginal births (data available for 71 countries) and from 2.5 days to 9.3 days for cesarean-section deliveries (data available for 30 countries). The UK was the high-income country with the shortest mean length of stay for singleton vaginal births. The percentage of stays that were “too short” ranged from 0.2% to 83% for vaginal births and from 1% to 75% for cesarean-section deliveries. In half of the 30 DHS countries, more than 20% of women who delivered in health facilities stayed too short.

Using a systematic approach to analyze the DHS results, the researchers then examined factors that influenced the length of stay. Longer stays because of increased need for care were found to be linked to birth by cesarean section, low birthweight, multiple births, and death of the newborn before discharge. Women delivered by doctors also had generally longer lengths of stay, as did older and poorer women.

**What Do these Findings Mean?:**

For many countries, the researchers were unable to find information on postpartum length of stay, and additional research seems warranted. Moreover, in the 30 DHS countries included in the analysis here, the percentage of births in health facilities ranged from 25.1% to 99.2%, suggesting that many countries still need to increase facility births. The postpartum lengths of stay reported vary widely between countries and are likely influenced by national norms and health system features in addition to specific needs of mothers and newborns. The reasons why women leave or are discharged early appear complex and are not well understood. Nonetheless, it seems that many women stay too short to receive adequate postnatal care. This is alarming, especially in low-income settings, where access to care after discharge is often limited. Countries in which staying too short is common should examine the reasons, clearly define appropriate care in health facilities during and after birth, and ensure both that such care is offered and that mothers and newborns stay long enough to receive it.

**Additional Information:**

Please access these websites via the online version of this summary at http://dx.doi.org/10.1371/journal.pmed.1001972.

- WHO provides recommendations on postnatal care of mothers and newborns
- WHO’s Partnership for Maternal, Newborn & Child Health has information on global maternal and child health
- The UK National Health Service has a pregnancy and baby guide that includes information on newborn health, postnatal check-ups, the mother’s body after birth, and breastfeeding
- The US Centers for Disease Control and Prevention has pages on maternal and infant health and global maternal and child health

## Introduction

Global efforts to prevent maternal and perinatal mortality aim to ensure all women have access to skilled attendants for childbirth, which in practice is virtually synonymous with advocating for facility delivery rather than home birth. Some countries, for example Malawi and Hungary, have even mandated that all births take place in facilities [1,2]. This skilled-birth-attendant/facility-delivery strategy stems from a recognition that most potentially fatal complications cannot be prevented or predicted, and that delivery and the first 24 h postpartum are the highest risk periods for women and newborns [3]. This strategy aims to ensure that women and newborns are situated in a place where emergency care can be provided, if needed, by a skilled health provider. Given the epidemiologic risk profile, and the efforts that many women and their families undertake to reach facilities in the first instance, it seems ill-considered not to guarantee that they, and their newborns, remain in facilities long enough to be adequately monitored and treated. Indeed, the World Health Organization (WHO) recommends that all women stay in facilities at least 24 h postpartum, albeit based on weak evidence [4]. Despite this recommendation, we suspect that women in many settings leave or are made to leave facilities quickly, before key postpartum checks can take place. Also, most low- and middle-income countries that have attempted to introduce postnatal home visitation programmes to complete these postpartum checks, or as an alternative when women deliver at home, have struggled to achieve more than low coverage [5].

We do not expect a uniform length of stay after delivery for all women or newborns; those with complications or greater vulnerability will typically require longer admissions, unless they die or are referred elsewhere. However, while appropriate stays will vary in length, some stays will be “too short” or “too long” relative to actual need. These stays may lead to adverse health outcomes, dissatisfaction, or increased costs. Specifically, short lengths of stay can leave insufficient time to detect, diagnose, or treat complications, which can in turn increase morbidity and mortality [6–10]. Insufficient time to educate or support women within facilities can also reduce maternal confidence or cause breastfeeding problems, maternal depression, or dissatisfaction with care [11–15]. Long lengths of stay can increase exposure to adverse facility environments, with increased risk of nosocomial infections, sleep disturbance, or poor infant-feeding support [12,16]. These can decrease maternal confidence, paternal involvement, or family bonding. They can also increase sibling rivalry, breastfeeding problems, or maternal dissatisfaction [17]. Furthermore, both inappropriately long lengths of stay and premature discharge leading to re-admission [18] are inefficient and increase financial costs to families and health systems [17,19,20].

There is a substantial literature on the determinants of length of stay in general. Schorr [21] reviewed it and proposed a model of the determinants of length of stay with four categories: patient characteristics, clinical caregiver characteristics, characteristics of the social or family environment, and characteristics of the health care system. The literature on length of stay following childbirth focuses on high-income countries, with a Cochrane review noting the paucity of data from low- and middle-income countries [15].

Our paper describes length of stay following childbirth in facilities and its main associated factors. The objectives are to identify and compile all available multi-country tabulations or reports on length of stay from multi-country population-based surveys, and to analyse the Demographic and Health Surveys (DHS) to estimate length of stay. Another objective is to use the literature to build a conceptual framework of the main determinants of length of stay and to analyse the DHS to understand the factors associated with length of stay and with the proportions of stays that are “too short” following vaginal and cesarean-section birth.

## Methods

We did not have identifiable data, and obtained ethical approval from the London School of Hygiene & Tropical Medicine Ethics Committee for our analyses (approval number 7190). The surveys analysed all sought country-government permission, obtained informed consent from respondents, and assured confidentiality.

We searched multi-country statistical tabulations of indicators including those by the Organisation for Economic Co-operation and Development (OECD) [22], Euro-Peristat (http://www.europeristat.com/), the Global Health Data Exchange (http://ghdx.healthdata.org/), Gapminder World (http://www.gapminder.org/data/), and UNdata (http://data.un.org/), which includes indicators from WHO, the United Nations Children's Fund, the United Nations Population Fund, and the World Bank, among others.

We also searched multi-country survey programmes likely to provide and or report data on length of stay for nationally representative samples. These included the DHS, the Multiple Indicator Cluster Surveys (MICS), the Centers for Disease Control and Prevention Reproductive Health Survey (CDC-RHS), and the Pan Arab Project for Family Health [22–25].

### Multi-country Tabulated Data or Reports

We identified multi-country indicator tabulations on length of stay from the OECD for 40 countries. We identified two survey programmes, namely MICS and CDC-RHS, that reported relevant data on 21 countries and one country, respectively. The DHS did not report on length of stay, but we used its electronically available datasets to conduct our own analyses for 30 countries [20,23–25]. When countries had multiple sources of estimates, we preferentially used the DHS because we could use the raw data to conduct our own analyses. If there were multiple surveys within a survey programme, we selected the most recent.

### Organisation for Economic Co-operation and Development

The OECD is a forum for governments that asks governments to report on, and sets standards for, a wide range of indicators, and analyses and compares data to predict future trends. Its indicators include length of stay following vaginal singleton delivery [20,22]. We searched all OECD tabulations of this indicator and identified the 40 countries reporting data for at least one year since 2000. Where indicators were tabulated for more than one year, we selected the most recent.

### Multiple Indicator Cluster Surveys

MICS are standardised, cross-sectional, nationally representative household surveys, covering an average of 11,000 households per country. Recent MICS country reports include the percentage of all facility births (typically in the 2 y before the survey) with a length of stay < 12 h. This is not stratified by mode of delivery or by singleton versus multiple (twin or triplet) status. Some countries also report on the percentage staying 12–23 h, totalling to the percentage staying <24 h. We reviewed all reports from the most recent MICS (2000 to 2015), and identified 21 countries with length-of-stay data that were not also captured via DHS [26]. Many MICS datasets are not in the public domain and so were not accessible to us.

### Centers for Disease Control and Prevention Reproductive Health Surveys

The CDC-RHS consists of cross-sectional, nationally representative household surveys largely focused on Latin America and Eastern Europe. Since the late 1980s, questions included in the CDC-RHS are comparable with those in the DHS; however, the CDC-RHS is not provided in a standardised format, and many of its surveys are not in the public domain. Summaries, and in some cases datasets and reports, for all surveys are available on the Global Health Data Exchange (http://ghdx.healthdata.org/series/reproductive-health-survey-rhs). We reviewed the summaries and reports and identified one survey with appropriately tabulated data (although several others had also asked about length of stay).

### Demographic and Health Surveys Data

The DHS are standardised, cross-sectional, nationally representative household surveys, usually covering 5,000 to 30,000 households [27]. Of relevance to our analyses, they measure length of stay, household and individual characteristics, child survival, and maternal health care use. We reviewed the most recent DHS (2000 to mid-2013) and identified 30 countries with length-of-stay data (S1 Table). If there were multiple surveys for a country, we selected the most recent.

### Length of Stay

Women in MICS who delivered a live birth in a facility in the 2 y before the survey were asked, “You have said that you gave birth in [name or type of facility]. How long did you stay there after the delivery?” Responses were recorded in hours if less than 1 d, days if less than 1 wk, and weeks otherwise.

Women in the CDC-RHS who delivered a live birth in a facility in the 5 y before the survey were asked, “How many nights were you in that place after delivery?” Responses were recorded as number of nights for 0 to 84 nights, “85+” for 85 or more nights, or “88” for “Don’t know” or “Don’t remember”.

Women in the DHS who delivered a live birth in a facility in the 5 y (or 3 y) before the survey were asked, “How long after [name] was delivered did you stay there?” Responses were recorded in hours if less than 1 d, days if less than 1 wk, and weeks otherwise. We converted responses recorded in days or weeks into hours, assuming stays ended at the mid-point of the day or week, respectively (for example, a stay of 1 d could have been 24–47 h, and so was assumed to be 36 h. We saw many responses ranging from 1 to 23 h, which suggests that a response of 1 d was at least 24 h or longer, up to 47 h, after which we assumed women would have reported staying 2 d, although we cannot rule out reporting error. Similarly, a stay of 1 wk was assumed to be 10.5 d, or 252 h. We analysed the responses for the most recent birth in the survey recall period.

Length-of-stay data are frequently right-skewed, with extreme outliers [28]. Our data were right-skewed, and some outliers were so extreme that we judged them to be errors in the unit of reporting (number of days incorrectly reported as number of weeks). We were also concerned that including these values would make it difficult to show the main distribution in the descriptive data on a linear scale and that some extreme outliers would be influential in the linear regression. It is also possible that the factors associated with staying an extreme length of time were different from those associated with an increased or decreased length of stay within more normal limits. In the logistic regression, these extreme outliers were not relevant. Based on our knowledge of guidelines on length of stay, we decided that 3 wk (equivalent to 588 h in our conversion) was a reasonable cutoff for extreme values and excluded observations with lengths of stay greater than 3 wk (584 observations, 0.5% of the unweighted sample). The influence of any remaining skewness was assessed in a sensitivity analysis that log-transformed the data.

### Conceptual Framework and Determinants

Conceptual frameworks lay out the key factors, constructs, or variables, and the relationships among them [29]. We developed our framework iteratively, using our technical knowledge and research background, a literature review of related theory and research, and concepts used previously to represent similar problems [30]. We classified and organised the concepts influencing length of stay to emphasise connections between them, connecting the main variables with each other, in a presumed relationship [29,30]. We used the framework to identify the determinants available for analysis within our DHS dataset and to group them into broad categories: need-related characteristics, facility/provider (health system) characteristics, women’s characteristics, and child-related characteristics. The main factors available to analyse, together with sub-categories used, are shown in the tables and S1 Text.

### Statistical Analysis

We used Stata (version 14.0; Stata Corporation, College Station, Texas, US) to conduct the analyses. The complex, multi-level cluster survey design used in the DHS, which often oversamples certain areas, was accounted for in the analysis by applying individual women’s survey weights that allowed for the survey design and non-response. These survey weights “weight down” women who are from areas that have been oversampled and “weight up” women from areas that have been undersampled. Similarly, when we present proportion estimates for the entire DHS sample (with countries pooled), the countries that have been oversampled are “weighted down”, and those undersampled “weighted up”, to get estimates that represent the proportion of women across the 30 DHS countries surveyed.

We used violin plots [31] and Kaplan-Meier graphs to explore the distribution of length of stay, and we estimated the proportions of women in each category pooled across countries, weighting according to country populations in 2008. Within each category, we estimated the mean and median length of stay.

### Linear regression

We performed a linear regression for each factor in turn using length of stay as the outcome, adjusting only for country. To understand how the rate of cesarean section influenced each of these odds ratios, we repeated the analyses adjusting for mode of delivery. Next, we performed a multivariable linear regression including all factors or potential confounders. Finally, we ran analyses stratified by mode of delivery. Results are expressed as the expected increase (or decrease) in length of stay in hours between the category and the baseline reference category, along with its 95% confidence interval.

### Logistic regression

We ran two sets of logistic regression analyses to identify factors associated with lengths of stay that were “too short”, defined as <24 h for vaginal and <72 h for cesarean-section births. The 24-h cutoff was based on a WHO recommendation for uncomplicated vaginal deliveries, but there was less basis for choosing a cesarean-section cutoff. We chose 72 h because the United States deemed stays of 48–72 h for cesarean section problematic and legislated that women be allowed to stay at least 96 h [11,32]. The model-building process was the same for the logistic regression as for the linear regression, and results are expressed as odds ratios comparing each category to the baseline reference category. We could not assess the proportion of women who stayed “too long”, as to do so would have required much better data on need-related characteristics (in terms of information on complications in the mother or newborn) than were available to us.

Each of the variables included in the data analysis was checked for missing observations, and the number of missing values recorded. For the regression analyses, a complete case analysis was performed, excluding all observations that had either the outcome or any of the exposure variables missing. However, if any individual variables had greater than 1% missing data or the total proportion of excluded observations exceeded 5%, then a sensitivity analysis was performed to identify whether excluding these records influenced the estimates obtained.

## Results

The percentage of births occurring in facilities ranged from 25.1% to 99.2% across the DHS countries, with the median at 72.1%. In these country surveys, 107,128 women reported having a live birth delivered in a facility within the last 5 y. Of these, 410 (0.4%) had a missing value for length of stay, and 584 (0.5%) were excluded because they reported a length of stay greater than 3 wk. So, the final DHS analysis sample of facility births included 106,134 (75,636 in the weighted sample) women. S1 Fig. shows a flow diagram of inclusions and exclusions for the sample. S1 Table shows, by country, the numbers and percentages of respondents with a birth in the last 5 y in a facility, missing and outlying lengths of stay, mean and median lengths of stay, and percentages of women staying too short by mode of delivery.

**Fig 1:**
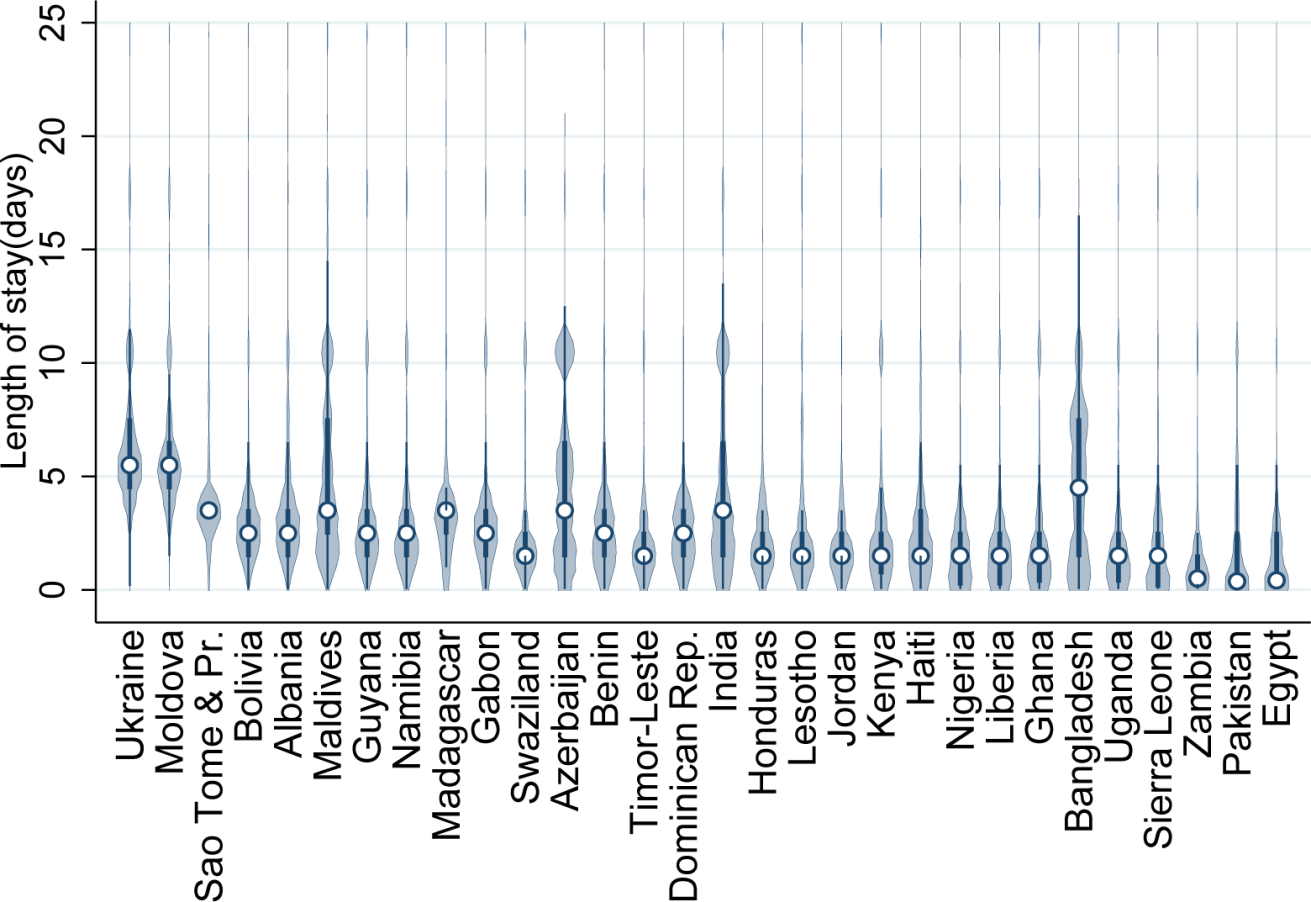
Violin plots of length of stay (in days) for all women, for 30 countries with DHS data. This figure illustrates the probability distribution of length of stay for each country, with the median indicated by a circle. Dominican Rep., Dominican Republic; Moldova, Republic of Moldova; Sao Tome & Pr., Sao Tome and Principe.

Fig 1 shows violin plots of length of stay for all women by DHS country, ordered by the percentage of women who stayed <24 h, lowest to highest. It indicates wide variability between and within countries, and skewed data. S2 Fig shows that length of stay in countries in the OECD tabulations has been decreasing over time.

**Fig 2:**
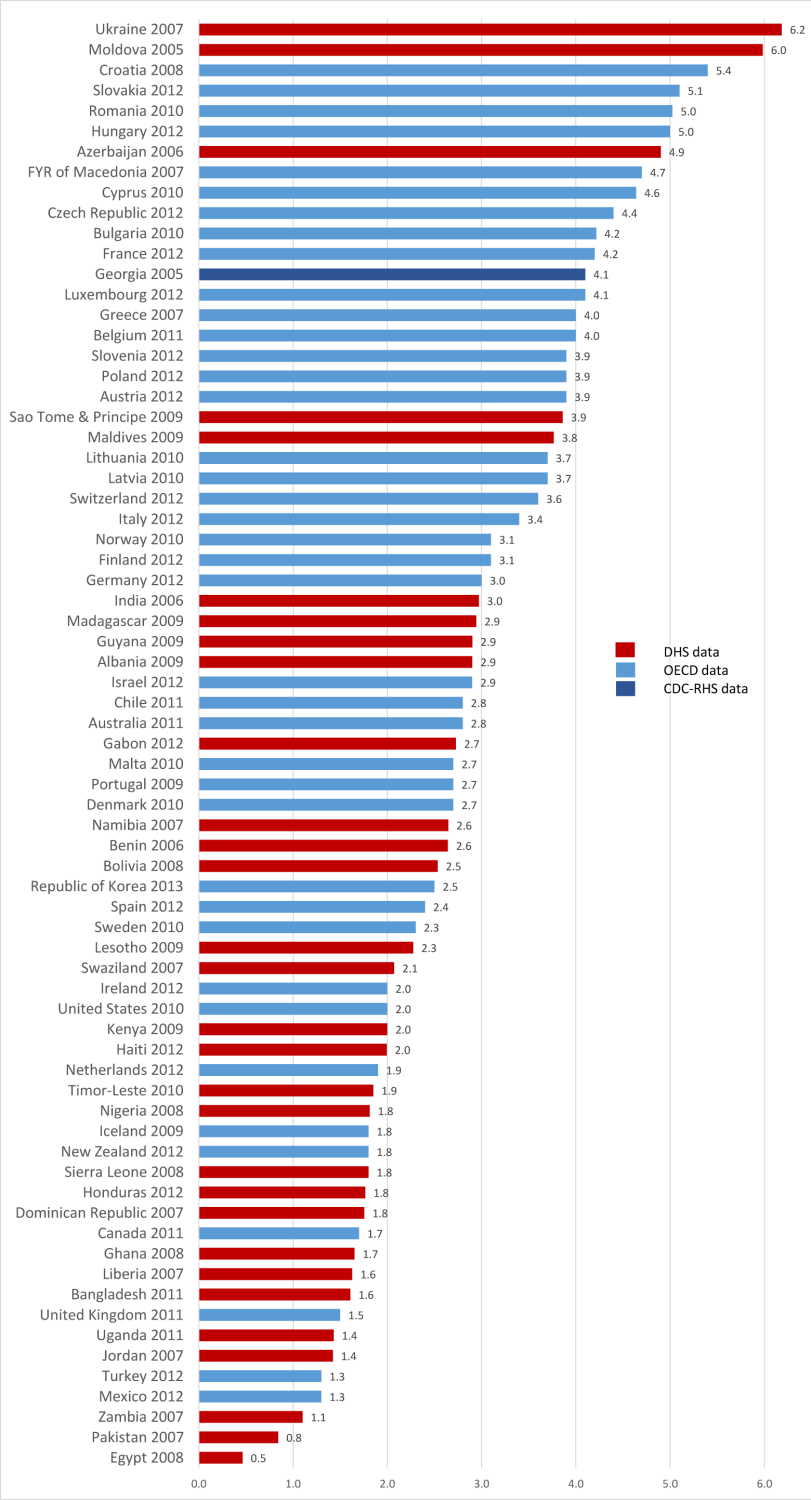
National-level data from DHS, OECD, and CDC-RHS on average length of stay after singleton vaginal delivery for 71 countries. FYR of Macedonia, former Yugoslav Republic of Macedonia; Moldova, Republic of Moldova.

**Fig 3:**
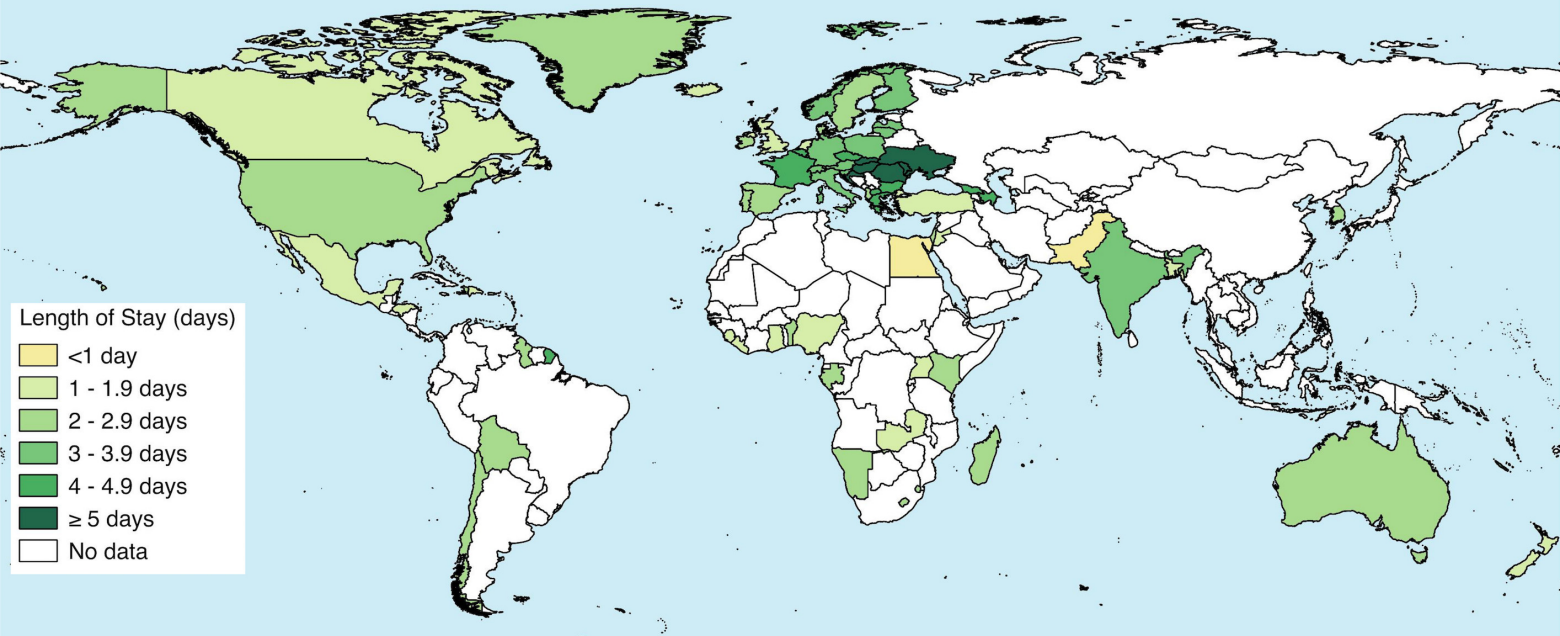
Map of countries with national-level data on length of stay after singleton vaginal deliveries. Data from OECD, DHS, and CDC-RHS.

Fig 2 shows the mean length of stay for vaginal singleton births from the DHS (30 countries) as well as from 41 further nationally representative sources (OECD, 40 countries, and CDC-RHS, one country). Fig 3 shows a map of countries with data by their average length of stay for vaginal singleton deliveries.

**Fig 4:**
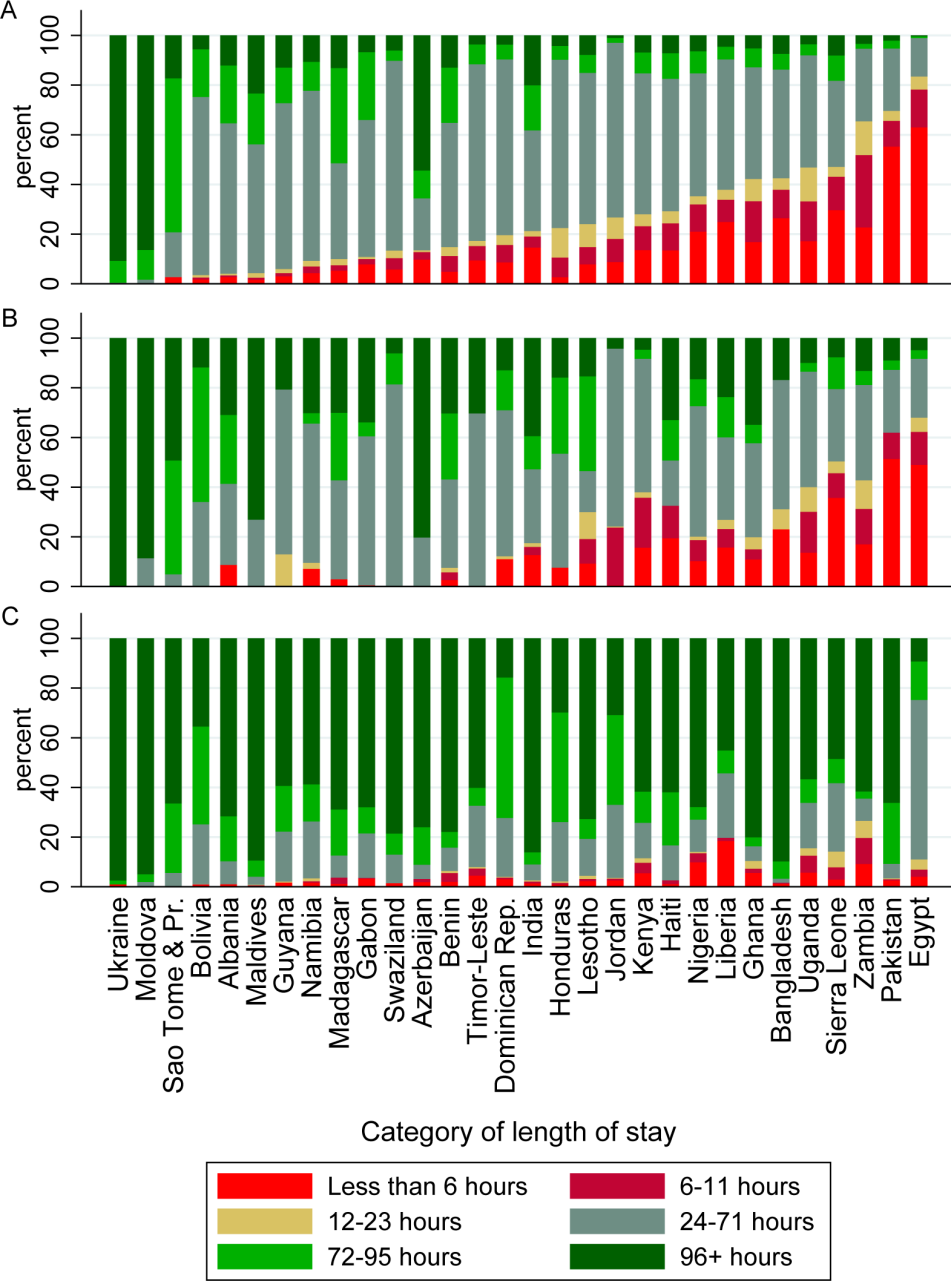
Proportion of vaginal singleton, vaginal multiple, and cesarean deliveries by category of length of stay, for 30 countries with DHS data. (A) Vaginal singleton deliveries; (B) vaginal multiple deliveries; (C) cesarean deliveries. Dominican Rep., Dominican Republic; Moldova, Republic of Moldova; Sao Tome & Pr., Sao Tome and Principe.

Fig 4A shows length of stay for vaginal singleton deliveries by DHS country, ordered by the percentage of women who stayed <24 h. Many countries have a substantial proportion of women with stays that may be too short. Fig 4B and 4C show length of stay for vaginal multiple and cesarean-section deliveries, respectively. S3 Fig. shows the proportion of all women staying <12 h (and 12–23 h where available), as abstracted from MICS reports for 21 countries and as estimated in the DHS for 30 countries.

**Fig 5:**
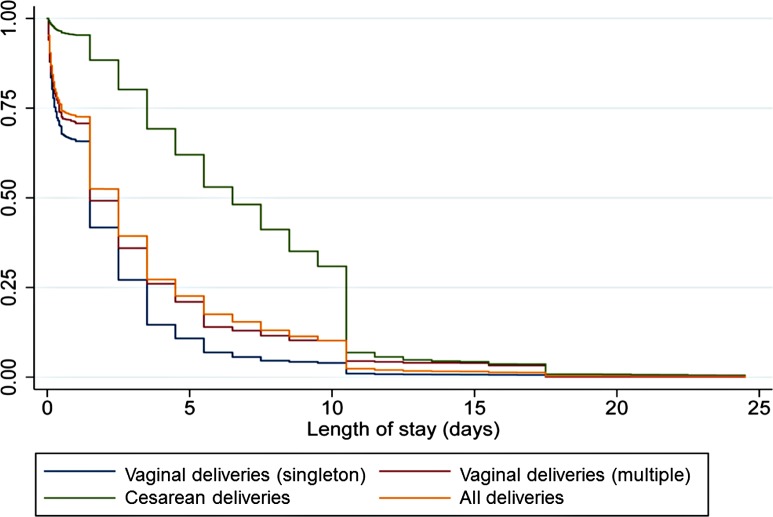
Kaplan Meier survival graphs for all women, by mode of delivery, and by multiplicity for vaginal deliveries. Data from DHS.

Fig 5 presents Kaplan-Meier survival graphs for all women in the DHS, by mode of delivery and multiplicity. Vaginal singleton deliveries had the shortest length of stay, followed by vaginal multiple deliveries and cesarean-section deliveries.

**Fig 6:**
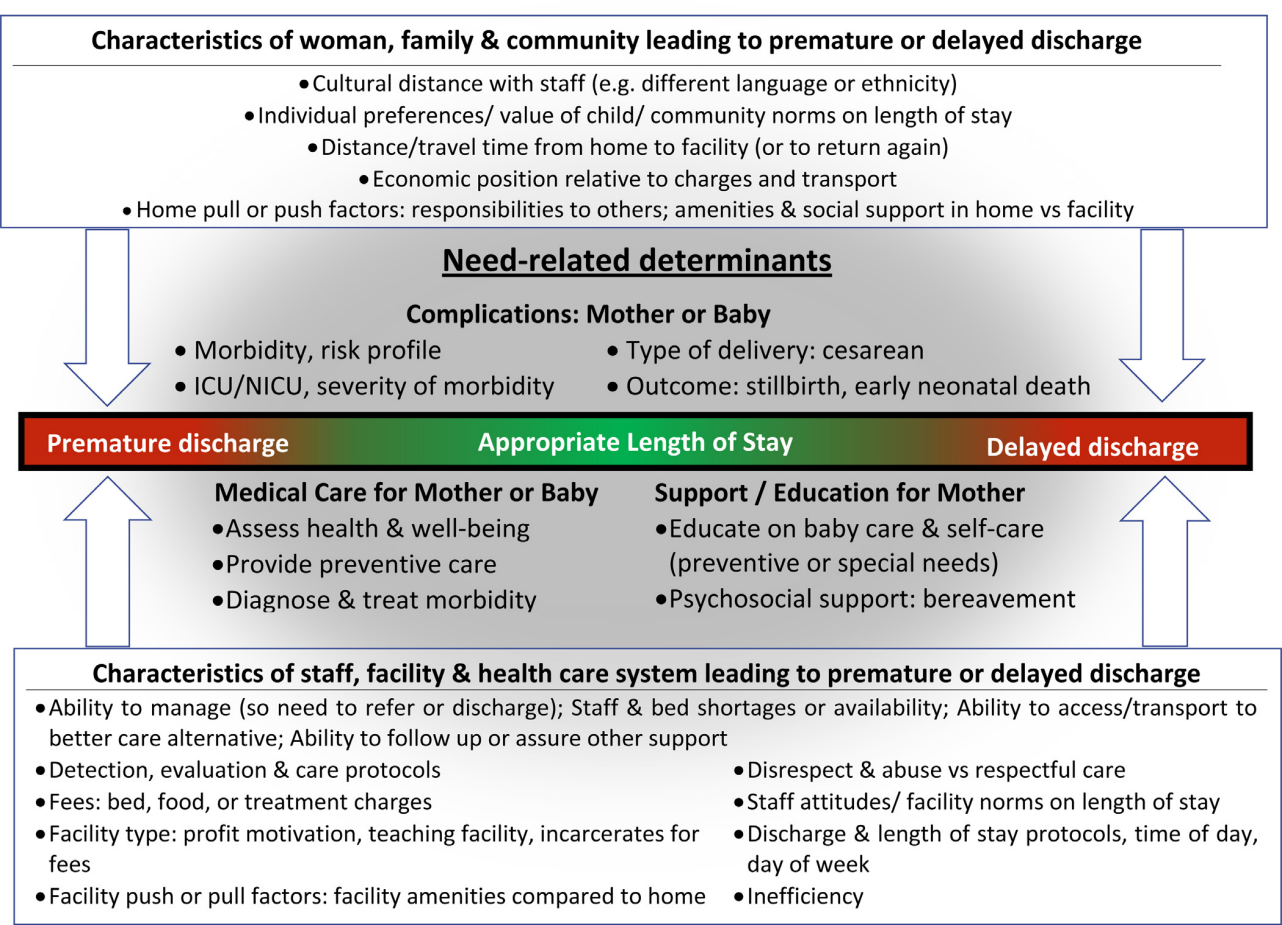
Conceptual framework. ICU, intensive care unit; NICU, neonatal intensive care unit.

Our conceptual framework (Fig 6) separated drivers of length of stay related to genuine need (“need-related characteristics”) from characteristics of women, their families, or their community (“women’s characteristics” and “child-related characteristics”), as well as characteristics of health facilities, their staff, and the health system (“facility/provider-related characteristics”). It suggested that the drivers of inappropriate lengths of stay, whether too short or too long, mirrored each other. For example, women who had other children at home, but no social support to look after them, might have had reasons to leave a facility early. By contrast, having plenty of social support might have facilitated a longer length of stay. The framework also made it possible to see that many drivers of length of stay were not measured in our data.

Table 1 shows the distribution of factors in the DHS countries, and the mean and median length of stay by factor. In general, there were few missing data, with eight out of the 14 factors listed having no missing values at all. Only birthweight had a substantial proportion of missing data, with 20,226 missing observations (19%), with the next highest being mode of delivery, which had 141 missing observations (0.1%).

**Table 1:** Distribution of factors in the DHS countries, and the mean and median length of stay by factor.

Category	Factor (Number of Missing Observations)	Percentage Women by Category	Length of Stay		Premature Discharge	
			Mean ± SD (Hours)	Median (IQR) (Days)	Vaginal Births: Percentage LoS < 24 h	Cesarean Births: Percentage LoS < 72 h
**Need-related characteristics**	**Mode of delivery (141)**					
	Vaginal	77.1%	56.1 ± 66.5	1.5 (1.0, 3.5)	33.7%	Not applicable
	Cesarean	22.9%	165.8 ± 101·3	4.5 (2.5, 8.5)	Not applicable	80.2%
	**Multiple birth (0)**					
	Singleton	98.3%	80.7 ± 88.2	2.5 (1.5, 3.5)	33.7%	19.7%
	Twins or triplets (2+)	1.7%	114.0 ± 112.7	3.5 (1.5, 5.5)	28.3%	17.1%
	**Birthweight (20,226)**					
	<1,999 g	4.6%	115.2 ± 115.6	3.5 (1.5, 6.5)	21.0%	18.4%
	2,000–2,499 g	11.3%	96.0 ± 87.8	2.5 (1.5, 3.5)	19.5%	14.8%
	2,500+ g	84.2%	89.8 ± 88.6	2.5 (1.5, 3.5)	24.0%	19.4%
	**Survival (index child) (0)**					
	Died before/on day of discharge	1.5%	111.7 ± 107.1	3.5 (1.5, 5.5)	23.4%	9.8%
	Survived	96.4%	81.0 ± 88.3	2.5 (1.5, 3.5)	33.5%	19.8%
	Died after discharge	2.1%	68.8 ± 92.9	1.5 (0.7, 3.5)	44.5%	22.6%
**Facility/provider characteristics**	**Birth attendant (68)**					
	Nurse-midwife	25.8%	49.6 ± 64.9	1.5 (0.8, 2.5)	38.2%	37.6%
	Doctor	72.1%	93.7 ± 93.6	2.5 (1.5, 4.5)	30.9%	18.3%
	Auxiliary staff/other	2.1%	41.4 ± 61.5	1.5 (0.3, 2.5)	47.0%	49.2%
	**Sector of facility (0)**					
	Public	51.6%	77.5 ± 88.5	2.5 (1.5, 3.5)	31.4%	19.9%
	Private	48.4%	85.2 ± 89.0	2.5 (1.5, 3.5)	36.4%	19.4%
**Women’s characteristics**	**Women’s age in years (0)**					
	15–19	6.4%	74.2 ± 83.5	1.5 (1.5, 3.5)	35.7%	17.9%
	20–24	29.1%	81.4 ± 86.0	2.5 (1.5, 3.5)	30.9%	19.1%
	25–29	32.5%	84.2 ± 90.4	2.5 (1.5, 3.5)	32.9%	19.4%
	30–34	18.7%	83.5 ± 91.0	2.5 (1.5, 3.5)	34.5%	20.1%
	35–39	9.2%	76.7 ± 90.9	2.5 (1.5, 3.5)	38.3%	20.7%
	40–44	3.3%	70.1 ± 87.0	2.5 (1.5, 3.5)	41.0%	21.8%
	45–49	0.8%	58.4 ± 84.0	2.5 (1.5, 3.5)	39.5%	28.1%
	**Residence (0)**					
	Rural	53.5%	76.7 ± 89.4	2.5 (1.5, 3.5)	37.8%	19.7%
	Urban	46.5%	86.4 ± 87.9	2.5 (1.5, 3.5)	28.5%	19.4%
	**Wealth quintile (0)**					
	Poorest	9.0%	64.7 ± 81.3	1.5 (1.5, 3.5)	40.1%	29.6%
	Poorer	13.7%	69.2 ± 85.2	2.5 (1.5, 3.5)	38.8%	25.7%
	Middle	19.9%	75.3 ± 87.9	2.5 (1.5, 3.5)	37.4%	22.5%
	Richer	25.5%	82.8 ± 90.3	2.5 (1.5, 3.5)	32.8%	20.7%
	Richest	32.7%	93.1 ± 90.1	2.5 (1.5, 3.5)	27.0%	15.4%
	**Completed education level (43)**					
	None	20.6%	63.0 ± 83.9	1.5 (0.5, 3.5)	44.9%	22.6%
	Primary	19.0%	68.1 ± 83.1	2.5 (1.5, 3.5)	36.4%	19.2%
	Secondary	46.0%	87.9 ± 90.5	2.5 (1.5, 3.5)	29.3%	20.0%
	Higher	14.4%	103.4 ± 89.8	2.5 (1.5, 4.5)	23.8%	17.0%
	**Marital status (5)**					
	Currently married	95.3%	81.5 ± 88.9	2.5 (1.5, 3.5)	27.6%	20.6%
	Never married	1.6%	73.6 ± 88.7	2.5 (1.5, 3.5)	33.9%	19.5%
	Formerly married	3.1%	76.0 ± 86.5	2.5 (1.5, 3.5)	29.9%	21.0%
**Child-related characteristics**	**Sex of child (0)**					
	Female	46.2%	80.4 ± 89.1	2.5 (1.5, 3.5)	33.4%	20.1%
	Male	53.8%	82.0 ± 88.6	2.5 (1.5, 3.5)	33.9%	19.0%
	**Birth order (index child) (0)**					
	1	33.8%	92.8 ± 91.7	2.5 (1.5, 3.5)	28.2%	17.7%
	2–3	44.8%	84.4 ± 89.7	2.5 (1.5, 3.5)	31.8%	19.6%
	4–6	16.7%	59.1 ± 78.6	1.5 (1.0, 3.5)	42.7%	27.2%
	7+	4.7%	47.1 ± 69.0	1.5 (0.6, 3.5)	48.0%	22.4%
	**Wantedness** ^a^ **(70)**					
	Wanted then	78.3%	83.6 ± 89.5	2.5 (1.5, 3.5)	32.5%	19.6%
	Wanted later	12.5%	75.3 ± 86.6	2.5 (1.5, 3.5)	36.3%	17.1%
	Wanted no more	9.2%	69.4 ± 84.9	2.5 (1.5, 3.5)	39.0%	23.5%

Results are weighted to allow for under-/oversampling of clusters and countries. Data from 30 DHS countries.

^a^Wantedness refers to whether the pregnancy resulting in the delivery under consideration was wanted at the time it occurred, was wanted but mistimed, or was not wanted at all.

IQR, interquartile range; LoS, length of stay; SD, standard deviation.

Table 2 shows crude associations between each factor and length of stay, adjusted for country and for country and cesarean section. Country effects are not displayed. The last column shows the final multivariable linear regression coefficients, adjusted for all variables in the model. The stratified analyses are in S2 Table.

**Table 2:** Linear regression models of the association between factors and length of stay in hours.

Category	Factor	RC (95% CI) Adjusted for Country	*p*-Value	RC (95% CI) Adjusted for Country and Mode of Delivery	*p*-Value	RC (95% CI) Adjusted for Country and All Other Covariates
**Need-related characteristics**	**Mode of delivery**		<0.001		<0.001	
	Vaginal	Reference		Reference		Reference
	Cesarean	84.0 (82.9; 85.1)		84.0 (82.9; 85.1)		80.4 (79.2; 81.6)
	**Multiple birth**		<0.001		<0.001	
	Singleton	Reference		Reference		Reference
	Twins or triplets (2+)	42.8 (39.3; 46.2)		29.1 (26.0; 32.1)		22.3 (18.8; 25.9)
	**Birthweight**		<0.001		<0.001	
	<1,999 g	44.4 (41.1; 47.0)		38.4 (36.8; 41.1)		34.3 (31.6; 36.9)
	2,000–2,499 g	8.8 (7.1; 10.5)		8.2 (6.6; 9.7)		6.9 (5.3; 8.4)
	2,500+ g	Reference		Reference		Reference
	**Survival (index child)**		<0.001		<0.001	
	Died before/on day of discharge	41.5 (37.3; 45.8)		32.1 (28.3; 35.9)		25.3 (19.8; 30.9)
	Survived	Reference		Reference		Reference
	Died after discharge	1.6 (−1.5; 4.7)		2.2 (−0.6; 4.9)		0.4 (−2.9; 3.8)
**Facility/provider characteristics**	**Birth attendant**		<0.001		<0.001	
	Nurse-midwife	Reference		Reference		Reference
	Doctor	43.0 (41.7; 44.3)		21.0 (19.8; 22.2)		19.8 (18.5; 21.2)
	Auxiliary staff/other	−3.1 (−0.1.; −6.1)		−3.0 (−5.7; −0.3)		−0.9 (−4.4; 2.6)
	**Sector of facility**		<0.001		<0.001	
	Public	Reference		Reference		Reference
	Private	6.6 (5.5; 7.7)		−3.6 (−4.6; −2.6)		−4.3 (−5.5; −3.1)
**Women’s characteristics**	**Woman’s age (years)**		<0.001		<0.001	
	15–19	−0.2 (−2.1; 1.6)		1.7 (0.0; 3.4)		−0.4 (−2.3; 1.5)
	20–24	Reference		Reference		Reference
	25–29	2.4 (1.2; 3.7)		0.7 (−0.4; 1.7)		2.5 (1.2; 3.7)
	30–34	6.4 (5.0; 7.7)		2.2 (1.0; 3.4)		4.9 (3.4; 6.3)
	35–39	9.0 (7.5; 10.6)		4.2 (2.8; 5.6)		7.5 (5.7; 9.3)
	40–44	12.5 (10.4; 14.7)		7.7 (5.8; 9.6)		11.7 (9.2; 14.2)
	45–49	13.5 (9.5; 17.4)		10.5 (7.0; 14.0)		16.3 (11.9; 20.7)
	**Residence**		<0.001		0.217	
	Rural	Reference		Reference		Reference
	Urban	4.0 (3.0; 4.9)		−0.5 (−1.3; 0.3)		−0.3 (−1.3; 0.8)
	**Wealth quintile**		<0.001		<0.001	
	Poorest	Reference		Reference		Reference
	Poorer	0.5 (−1.1; 2.0)		−2.5 (−3.9; −1.2)		−2.3 (−3.8; −0.8)
	Middle	0.9 (−0.6; 2.4)		−4.2 (−5.5; −2.8)		−5.3 (−6.8; −3.7)
	Richer	4.2 (2.7; 5.7)		−4.3 (−5.6; −2.9)		−5.9 (−7.5; −4.2)
	Richest	10.8 (9.3; 12.3)		−3.8 (−5.1; −2.4)		−7.0 (−8.9; −5.2)
	**Completed education level**		<0.001		<0.001	
	None	Reference		Reference		Reference
	Primary	6.5 (4.9; 8.0)		4.8 (3.4; 6.2)		4.3 (2.6; 6.0)
	Secondary	10.3 (8.9; 11.8)		3.8 (2.5; 5.1)		4.0 (2.2; 5.7)
	Higher	18.5 (16.7; 20.4)		2.2 (0.6; 3.9)		1.8 (−0.3; 4.0)
	**Marital status**		<0.001		<0.001	
	Currently married	Reference		Reference		Reference
	Never married	4.0 (2.0; 6.1)		4.6 (2.8; 6.4)		4.0 (2.0; 6.1)
	Formerly married	2.4 (0.6; 4.2)		4.0 (2.4; 5.7)		3.1 (1.3; 4.8)
**Child-related characteristics**	**Sex of child**		<0.001		0.018	
	Female	Reference		Reference		Reference
	Male	1.9 (1.0; 2.8)		0.9 (0.2; 1.7)		1.4 (0.6; 2.3)
	**Birth order (index child)**		<0.001		<0.001	
	1	Reference		Reference		Reference
	2–3	−4.3 (−5.4; −3.3)		−2.1 (−3.1; 1.2)		−3.9 (−5.1; −2.8)
	4–6	−7.4 (−7.8; −6.1)		−0.2 (−1.4; 0.9)		−4.7 (−6.4; −3.0)
	7+	−4.7 (−6.6; −2.7)		1.7 (0.0; 3.5)		−6.6 (−9.2; −3.9)
	**Wantedness**		<0.001		<0.001	
	Wanted then	Reference		Reference		Reference
	Wanted later	−1.8 (−3.0; −0.6)		0.7 (−0.4; 1.7)		1.1 (0.0; 2.3)
	Wanted no more	−1.5 (−2.9; −0.1)		2.9 (1.7; 4.2)		2.0 (0.6; 3.5)

Regression coefficients, in hours, with *p-*values and 95% confidence intervals, for models adjusted for country and for country and mode of delivery (cesarean versus vaginal), and for the final multivariable linear regression model adjusted for country and all other covariates. Data from 30 DHS countries.

CI, confidence interval; RC, regression coefficient.

Table 3 displays the factors associated with having a length of stay that is too short (premature discharge) based on logistic regression models adjusted for country, and multivariable logistic regression models adjusted for country and all other factors.

**Table 3:** Factors associated with lengths of stay that are too short.

Category	Factor (Missing Values for Vaginal and Cesarean Deliveries)	Vaginal Births: Length of Stay < 24 h			Cesarean Births: Length of Stay < 72 h		
		OR (95% CI) Adjusted for Country	*p*-Value	OR (95% CI) Adjusted for All Other Covariates (*n =* 68,562)	OR (95% CI) Adjusted for Country	*p*-Value	OR (95% CI) Adjusted for All Other Covariates (*n =* 17,604)
**Need-related characteristics**	**Multiple birth (0; 0)**		<0.001			<0.001	
	Singleton	Reference		Reference	Reference		Reference
	Twins or triplets (2+)	0.55 (0.47; 0.64)		0.59 (0.48; 0.72)	0.51 (0.41; 0.65)		0.57 (0.44; 0.75)
	**Birthweight (16,469; 3,822)**					<0.001	
	<1,999 g	0.82 (0.71; 0.93)	<0.001	0.86 (0.75; 0.99)	0.59 (0.48; 0.73)		0.69 (0.54; 0.83)
	2,000–2,499 g	0.86 (0.80; 0.93)		0.88 (0.81; 0.95)	0.77 (0.66 0.88)		0.81 (0.70; 0.94)
	2,500+ g	Reference		Reference	Reference		Reference
	**Survival (index child) (0; 0)**					<0.001	
	Died before/on day of discharge	0.54 (0.44; 0.65)	<0.001	0.54 (0.39; 0.75)	0.43 (0.30; 0.61)		0.33 (0.19; 0.59)
	Survived	Reference		Reference	Reference		Reference
	Died after discharge	1.11 (0.99; 1.24)		0.98 (0.84; 1.13)	0.87 (0.65; 1.15)		0.96 (0.69; 1.35)
**Facility/provider characteristics**	**Birth attendant (55; 14)**		<0.001				
	Nurse-midwife	Reference		Reference	Variable not applicable		Variable not applicable
	Doctor	0.60 (0.57; 0.63)		0.64 (0.61; 0.69)			
	Auxiliary staff/other	1.29 (1.17; 1.42)		1.22 (1.07; 1.41)			
	**Sector of FACILITY (0; 0)**		0.047			<0.001	
	Public	Reference		Reference	Reference		Reference
	Private	0.96 (0.92; 1.00)		1.02 (0.97; 1.08)	1.58 (1.47; 1.72)		1.51 (1.37; 1.65)
**Women’s characteristics**	**Women’s age in years (0; 0)**		0.006			<0.001	
	15–19	1.00 (0.93; 1.08)		1.00 (0.92; 1.10)	0.94 (0.80; 1.10)		1.00 (0.83; 1.19)
	20–24	Reference		Reference	Reference		Reference
	25–29	0.97 (0.93; 1.02)		0.96 (0.91; 1.02)	1.01 (0.92; 1.12)		0.99 (0.88; 1.11)
	30–34	0.95 (0.90; 1.00)		0.91 (0.84; 0.97)	0.87 (0.78; 0.96)		0.81 (0.71; 0.92)
	35–39	0.92 (0.86; 0.98)		0.86 (0.79; 0.94)	0.82 (0.72; 0.93)		0.76 (0.65; 0.89)
	40–44	0.88 (0.81; 0.96)		0.80 (0.71; 0.90)	0.70 (0.59; 0.84)		0.69 (0.56; 0.86)
	45–49	0.83 (0.72; 0.97)		0.65 (0.52; 0.81)	0.50 (0.34; 0.73)		0.42 (0.27; 0.68)
	**Residence (0; 0)**		<0.001			0.017	
	Rural	Reference		Reference	Reference		Reference
	Urban	0.87 (0.84; 0.91)		0.98 (0.93; 1.02)	1.09 (1.02; 1.18)		1.02 (0.93; 1.12)
	**Wealth quintile (0; 0)**		<0.001			0.001	
	Poorest	Reference		Reference	Reference		Reference
	Poorer	0.99 (0.93; 1.05)		0.99 (0.92; 1.06)	1.08 (0.95; 1.24)		1.08 (0.93; 1.26)
	Middle	1.02 (0.97; 1.09)		1.07 (0.99; 1.15)	1.20 (1.05; 1.36)		1.16 (0.99; 1.35)
	Richer	0.97 (0.91; 1.02)		1.08 (1.00; 1.17)	1.37 (1.21; 1.55)		1.36 (1.16; 1.59)
	Richest	0.79 (0.75; 0.84)		1.03 (0.95; 1.13)	1.37 (1.21; 1.55)		1.37 (1.15; 1.62)
	**Completed education level (25; 19)**		<0.001			0.084	
	None	Reference		Reference	Reference		Reference
	Primary	0.84 (0.79; 0.89)		0.95 (0.88; 1.02)	0.91 (0.78; 1.06)		0.80 (0.66; 0.96)
	Secondary	0.77 (0.73; 0.82)		0.97 (0.90; 1.05)	0.95 (0.83; 1.09)		0.72 (0.60; 0.87)
	Higher	0.62 (0.57; 0.67)		0.88 (0.80; 0.98)	1.04 (0.90; 1.21)		0.73 (0.59; 0.90)
	**Marital status (4; 1)**		0.003			0.147	
	Currently married	Reference		Reference	Reference		Reference
	Never married	0.90 (0.83; 0.98)		0.91 (0.83; 1.01)	0.96 (0.81; 1.14)		1.00 (0.82; 1.20)
	Formerly married	0.91 (0.84; 0.97)		0.91 (0.84; 0.99)	0.88 (0.77; 1.00)		0.92 (0.80; 1.06)
**Child-related characteristics**	**Sex of child (0; 0)**		0.839			0.164	
	Female	Reference		Reference	Reference		Reference
	Male	1.0 (0.96; 1.03)		1.01 (0.97; 1.06)	1.05 (0.98; 1.13)		1.08 (1.00; 1.17)
	**Birth order (index child) (0; 0)**		<0.001			0.011	
	1	Reference		Reference	Reference		Reference
	2–3	1.11 (1.07; 1.17)		1.12 (1.06; 1.19)	0.92 (0.85; 1.00)		1.01 (0.92; 1.12)
	4–6	1.12 (1.13; 1.25)		1.20 (1.11; 1.30)	0.89 (0.80; 1.00)		1.12 (0.96; 1.31)
	7+	1.15 (1.07; 1.24)		1.23 (1.09; 1.39)	0.75 (0.62; 0.91)		1.07 (0.83; 1.40)
	**Wantedness (58; 12)**		<0.001			0.101	
	Wanted then	Reference		Reference	Reference		Reference
	Wanted later	1.14 (1.09; 1.19)		1.13 (1.07; 1.19)	1.03 (0.94; 1.13)		1.04 (0.94; 1.15)
	Wanted no more	1.06 (1.00; 1.12)		1.03 (0.97; 1.11)	0.90 (0.80; 1.01)		1.00 (0.88; 1.15)

Logistic regression for vaginal deliveries staying < 24 h (*n =* 85,124) and cesarean-section deliveries staying < 72 h (*n =* 21,453), showing odds ratios with 95% confidence intervals adjusted for country and for all other covariates. Data from 30 DHS countries.

CI, confidence interval; OR, odds ratio.

We conducted two types of sensitivity analyses (S4 Fig; S3 Table). In the first, we checked the residuals from the final linear regression model and found they were not normally distributed, so we repeated the linear regression using log-transformed length-of-stay outcomes. The results from this analysis provided estimates of increased/decreased length of stay of a similar magnitude to those in the original linear regression. We decided that the estimates of increase/decrease in length of stay in hours obtained from the original regression would be clearer to interpret. In the second sensitivity analysis, we checked how missing values affected the analysis. The linear regression performed was a complete case analysis (i.e., women with any of the included variables missing were excluded from analysis). However, birthweight was missing for 20,226 deliveries, including for all observations in Bangladesh, where this question was not asked. This led to 19% of the sample being excluded when birthweight was added to the model. We therefore performed the second sensitivity analysis excluding birthweight from the linear regression, and found that excluding it made little difference to the effect size estimates for any of the other factors. The effect of other missing data on the outcome was negligible, with only 184 individuals having one or more missing values (0.2%).

### Syntheses of Results

Across the DHS analyses and models, many of the associations were consistent, and, with few exceptions, the factors associated with longer lengths of stay in the linear regression were associated with smaller proportions of stays that were too short in the logistic regression for both modes of delivery (S2 Table). Cesarean-section mode of delivery was independently and consistently associated with longer lengths of stay, and had the largest effect size. Deliveries with other need-related factors (twins or triplets, lower birthweight, or an index child who died before or on the discharge day) were independently and consistently associated with longer lengths of stay in the linear regression, or with a lower proportion staying too short in the logistic analyses stratified by mode of delivery. Deliveries where infants died after discharge were associated with shorter average stays and more stays that were too short in the crude means and proportions, but the pattern reversed, such that death after discharge was associated with longer stays (although not always statistically significantly), once country was adjusted for. This reversal of the direction of association was because, on average, countries with higher infant mortality—and hence higher post-discharge mortality—had shorter lengths of stay.

### Facility/Provider-Related Characteristics

Doctor-attended deliveries were associated with longer lengths of stay and a lower proportion staying too short in all analyses. The subset of women whose infants were delivered by cesarean section are expected to have been attended largely, if not exclusively, by doctors, so we excluded birth attendant from this model. Women attended by non-skilled staff consistently had shorter lengths of stay than those attended by nurse-midwives. In the crude analyses, it appeared that women who delivered in private-sector facilities stayed longer than those who delivered in the public sector did. However, once we adjusted for cesarean mode of delivery or stratified by it, women in the private sector stayed shorter. The apparent association with longer stay in the crude analyses was due to a greater proportion of cesarean sections in the private sector. In the stratified analyses, the association of length of stay with sector was not significant among vaginal births, while among cesarean-section births, private-sector births were 50% more likely to be too short (<72 h) than public-sector births.

### Women’s Characteristics

Women’s characteristics independently and consistently associated with longer lengths of stay, or with a lower proportion staying too short, included older age and not being currently married. In the crude analyses of mean length of stay and proportion of women staying too short, the older the women were, the more likely they were to have a shorter or too short stay. Once country was adjusted for, this trend reversed, so older age became associated with longer stays, because countries with more births to older mothers usually had shorter lengths of stay. The same reversal in the direction of the association also occurred for women who were not currently married, although the association for not currently married women was not statistically significantly different from that of currently married women in the cesarean-section model. Having higher education was independently and consistently associated with a lower proportion staying too short in all analyses except those that accounted for cesarean-section delivery via adjustment (linear regression) or stratification (logistic regression). Urban residence also increased length of stay, but this crude association operated largely through higher cesarean-section rates in urban areas. Once mode of delivery was adjusted for, residence ceased to have a statistically significant association with length of stay. In the analysis stratified by mode of delivery, women with a cesarean-section delivery who lived in rural areas were less likely to stay too short than women who lived in urban areas.

In the crude analyses of mean length of stay and proportion staying too short, the wealthier the quintile, the longer women appeared to stay. However, once the linear regression was adjusted for cesarean section or stratified by mode of delivery, the pattern reversed and wealthier women stayed shorter. In the stratified analysis of women with cesarean-section deliveries, the odds of staying too short were 27% higher among the richest compared to the poorest quintile of women.

### Child-Related Characteristics

Women delivering infants of lower birth order or male infants stayed longer and were less likely to stay too short compared to women delivering infants of higher birth order or female infants. The association of birth order with length of stay was not significant in either of the stratified logistic analyses. The association of giving birth to a male child with length of stay was small and not significant in either stratified logistic regression. Women whose newborns were wanted stayed longer (and were less likely to stay too short) than those whose pregnancies were mistimed or unwanted in crude analyses. In the adjusted linear regressions, women with mistimed or unwanted pregnancies stayed slightly longer. Wantedness was not a significant factor associated with the proportion staying too short in the adjusted logistic regressions stratified by mode of delivery.

## Discussion

Our paper reports mean and median lengths of stay and proportions of stays that were too short (<24 h for vaginal deliveries and <72 h for cesarean-section deliveries) for up to 92 countries using OECD (40 countries), MICS (21 countries), CDC-RHS (one country), and DHS (30 countries) data. For singleton vaginal deliveries (71 countries), the mean length of stay ranged from 0.5 to 6.2 d for vaginal deliveries and from 2.5 to 9.3 d for cesarean deliveries. The proportion of women with vaginal deliveries who stayed too short (<24 h) ranged from 0.1% to 83.2%, while the proportion with cesarean-section deliveries who stayed too short (<72 h) ranged from 1.0% to 75.3% across the 30 DHS countries. In half of the 30 DHS countries, more than a fifth of women who delivered in facilities stayed too short. We developed a comprehensive conceptual framework that delineated the relationship between various factors and length of stay. Based on this framework, we quantified the association of selected factors with length of stay. In the 30 DHS countries, we found that the important factors associated with longer stays—including cesarean-section delivery, low birthweight, multiple births, and infants that did not survive beyond discharge—seemed to be linked largely to individuals who need greater care. Women delivered by doctors had longer lengths of stay, and women’s characteristics, particularly older age, played an important role in extending length of stay. The wealthier the women, the shorter their stays appeared to last.

### Limitations

Using existing tabulated data meant we relied on definitions used by others; further, in our secondary data analyses, we were unable to analyse all the potential determinants elaborated in the conceptual framework, particularly those related to medical need (of mother or newborn), facility features, providers, or the health system. The strengths of our DHS analyses were that they used nationally representative, comparable data for many countries in which, to our knowledge, length of stay after childbirth had not been studied previously. However, our DHS analyses also have limitations. Responses relied on women’s self-reports of their most recent live birth, recalled for up to 5 y, and stillbirths were excluded. Similarly, women who died, albeit a small percentage, were also excluded. The response option grouping of lengths of stay above 6 d into weeks resulted in heaping, because, for example, women staying between 7 to 13 d would have all been reported as staying 1 wk; our analysis considered them as staying 10.5 d (the mid-point of 7 to 13 d). This approach may have exaggerated the mean length of stay for cesarean sections or for countries at the longer end of the distribution. Also, some women identified non-doctor/non-clinical-officer individuals as their birth attendant for a cesarean section, an anomaly noted previously [33]. We are uncertain whether women misclassified their delivery provider or their mode of delivery, although the latter is thought to be well understood by women [34].

Until now, the main data available on mean length of stay were for vaginal singleton births compiled for 40 middle- and high-income countries by the OECD [20]. We conducted new analyses with 30 DHS datasets and compiled reports on a further 22 countries from MICS (21 countries) and the CDC-RHS (one country). Between-country variability was extremely large; for example, women with vaginal singleton deliveries stayed 12 times longer on average in the Ukraine than in Egypt. Country remained strongly associated with length of stay even after adjusting for many other variables, suggesting that national norms and health system features are important, beyond variations in case mix and consequent need. Norms that stipulate a minimum stay, for example, the 7 d required in the former Soviet Union [35], may explain the longer lengths of stays.

The more worrying finding was that in some countries many women left almost immediately, well before the 24 h recommended (the proportion staying <6 h ranged from 0.2% in Ukraine to 43.1% in Pakistan). Very short lengths of stay partially obviate the purpose of facility delivery, and seem perverse when so many initiatives seek to encourage postnatal checking of newborns, including by having health workers visit women’s homes up to three times in the first 10 d [36]. While some countries might mitigate the potential adverse consequences of early facility discharge through routine postnatal home visits by midwives after discharge [37,38], many do not. Countries with substantial proportions staying too short should seek to understand whether women leave so soon because of overcrowding, insufficient nursing support, or poor physical environments. Irrespective of reason, wide variations in length of stay are suggestive of potentially poor-quality care, possibly reinforced by a weak scientific evidence base on the optimal length of stay.

Nearly all the variables we explored were associated with length of stay in the expected direction, and the indicators of need or of complication had the largest associations, with cesarean-section mode of delivery having the single largest association. Women with a cesarean-section delivery residing in urban areas were more likely to stay too short compared to women residing in rural areas, possibly because those in remote areas received cesarean section for more severe indications and needed longer to recover. We were interested by the finding that the richest women stayed shorter than the poorest women did, because we expected women with greater financial resources to have fewer resource constraints and more options for attaining higher-quality care. The reasons wealthier women stayed shorter might include that they were healthier, operated with more self-efficacy, or were treated more efficiently by facility staff. For example, in some settings, women are detained until they pay facility fees [39]. Women with greater economic resources may have found arranging transport back home easier. Alternatively, they may have delivered in larger, higher-volume facilities that discharged women quickly to reduce overcrowding. Finally, our conceptual framework suggested that the balance of attractiveness between the home and facility environments may be a driver, and for the richest, the balance may favour the home environment [40].

## Conclusions

Substantial proportions of women stay too short following childbirth to allow for adequate postnatal care. Countries need to formulate coherent policy objectives to clarify what they intend to achieve with facility deliveries, including length of stay. Globally, women are encouraged to deliver in facilities in the first instance because this is the easiest place to provide skilled birth attendance and evidence-based postnatal care adhering to minimal global standards. Ensuring that such care is available, and that women and their newborns stay long enough to receive it, is essential. The challenge will be to identify the resources and commitment to increase lengths of stay that are too short, while also ensuring that any increase in time spent in facilities is used to actually provide appropriate-quality care. This will require greater resources and value than is currently being given to women’s and newborns’ health.

There is a need, at a minimum, to standardise the indicators used for multi-country comparisons with respect to stratification by mode of delivery, singleton versus all births, and cutoffs for stays considered too short. It may also be useful to conduct further research to measure variables not routinely available and look at determinants of length of stay within countries, where facility norms and community cultures are more similar.

## Supporting Information

S1 STROBE Checklist Completed STROBE checklist for observational studies. (DOC)

S1 Analysis Plan Broad and detailed analytical plans and changes made to these. (DOCX)

S1 Data List of sources of data used in the study. (DOCX)

S1 Fig Flow diagram of data for the DHS analysis. (TIF)

S2 Fig Lengths of stay over time from 1970 to 2012 in 23 selected OECD countries with data. (TIF)

S3 Fig Percentage of all deliveries (not stratified by mode of delivery) with length of stay <12 h and 12–23 h, totalling to percentage <24 h. Not all MICS report the percentage with length of stay 12–23 h. MICS include births up to 2 y before the survey; the DHS includes births up to 5 y before the survey.
(TIF)

S4 Fig Residuals from linear regression using untransformed and log-transformed outcomes. (TIF)

S1 Table DHS analysis. Number of women respondents per country, number and percentage (weighted using within-country sample weights) with a live birth in the 5 y before the survey, with at least one birth in a facility, and with a missing length of stay (LoS). Mean length of stay ± standard deviation (SD), in days; median length of stay (interquartile range); percentage of vaginal deliveries staying <24 h; percentage cesarean-section deliveries staying <72 h.
(DOCX)

S2 Table Heatmap showing direction of associations across all models in the DHS analysis. (DOCX)

S3 Table Sensitivity analyses: linear regression using log-transformed length-of-stay outcomes and linear regression excluding birthweight. (DOCX)

S1 Text Categorisation of factors examined in the DHS analysis. (DOCX)

## Abbreviations

CDC-RHS: Centers for Disease Control and Prevention Reproductive Health Survey
DHS: Demographic and Health Surveys
MICS: Multiple Indicator Cluster Surveys
OECD: Organisation for Economic Co-operation and Development
WHO: World Health Organization
