# Supplementary material for: Length of Stay After Childbirth in 92 Countries and Associated Factors in 30 Low- and Middle-Income Countries: Compilation of Reported Data and a Cross-sectional Analysis from Nationally Representative Surveys
Source: PLoS Med. 2016 Mar 8;13(3):e1001972. doi: 10.1371/journal.pmed.1001972 (PMC4783077; doi:10.1371/journal.pmed.1001972)
Supplement: S1 STROBE Checklist — (DOC) [file pmed.1001972.s001.doc]

STROBE Statement—checklist of items that should be included in reports of observational studies

|  | Item No | Recommendation | Location |
| --- | --- | --- | --- |
| **Title and abstract** | 1 | (*a*) Indicate the study’s design with a commonly used term in the title or the abstract | Title page |
|  |  | (*b*) Provide in the abstract an informative and balanced summary of what was done and what was found | Abstract |
| Introduction | | |  |
| Background/rationale | 2 | Explain the scientific background and rationale for the investigation being reported | Paragraphs 1,2 and 4 of Introduction |
| Objectives | 3 | State specific objectives, including any pre-specified hypotheses | Paragraphs 2-4 of Introduction |
| Methods | | |  |
| Study design | 4 | Present key elements of study design early in the paper | Paragraphs 1 and 2 of Methods |
| Setting | 5 | Describe the setting, locations, and relevant dates, including periods of recruitment, exposure, follow-up, and data collection | S1 Table, Fig 2, and S3 Fig |
| Participants | 6 | *Cross-sectional study*—Give the eligibility criteria, and the sources and methods of selection of participants | S1 Fig and paragraph 3 of Methods |
| Variables | 7 | Clearly define all outcomes, exposures, predictors, potential confounders, and effect modifiers. Give diagnostic criteria, if applicable | Paragraphs 3-5 of Methods and S1 Text |
| Data sources/ measurement | 8* | For each variable of interest, give sources of data and details of methods of assessment (measurement). Describe comparability of assessment methods if there is more than one group | Paragraph 3-5 of Methods and S1 Text |
| Bias | 9 | Describe any efforts to address potential sources of bias | Paragraph 1 of Discussion |
| Study size | 10 | Explain how the study size was arrived at | S1 Text |
| Quantitative variables | 11 | Explain how quantitative variables were handled in the analyses. If applicable, describe which groupings were chosen and why | Paragraphs 3-5 of Methods, S1 Text, and Table 1 |
| Statistical methods | 12 | (*a*) Describe all statistical methods, including those used to control for confounding | Paragraphs 7-11 of Methods |
|  |  | (*b*) Describe any methods used to examine subgroups and interactions | Paragraphs 7-11 of Methods |
|  |  | (*c*) Explain how missing data were addressed | Paragraph 11 of Methods, paragraph 1 of Results |
|  |  | *Cross-sectional study*—If applicable, describe analytical methods taking account of sampling strategy | Paragraph 7 of Methods |
|  |  | (*e*) Describe any sensitivity analyses | Paragraph 9 of Methods, paragraph 10 of Results |
| **Results** | | | |
| Participants | 13* | (a) Report numbers of individuals at each stage of study—eg numbers potentially eligible, examined for eligibility, confirmed eligible, included in the study, completing follow-up, and analysed | S1 Fig and S1 Table |
|  |  | (b) Give reasons for non-participation at each stage | S1 Fig |
|  |  | (c) Consider use of a flow diagram | S1 Fig |
| Descriptive data | 14* | (a) Give characteristics of study participants (eg demographic, clinical, social) and information on exposures and potential confounders | S1 Table and Table 1 |
|  |  | (b) Indicate number of participants with missing data for each variable of interest | Table 1 |
| Outcome data | 15* | *Cross-sectional study—*Report numbers of outcome events or summary measures | Table 1 |
| Main results | 16 | (*a*) Give unadjusted estimates and, if applicable, confounder-adjusted estimates and their precision (eg, 95% confidence interval). Make clear which confounders were adjusted for and why they were included | Table 2, Table 3, and S2 Table |
| Other analyses | 17 | Report other analyses done—eg analyses of subgroups and interactions, and sensitivity analyses | S2 Table |
| Discussion | | | |
| Key results | 18 | Summarise key results with reference to study objectives | Paragraph 1 of Discussion |
| Limitations | 19 | Discuss limitations of the study, taking into account sources of potential bias or imprecision. Discuss both direction and magnitude of any potential bias | Paragraph 2 of Discussion |
| Interpretation | 20 | Give a cautious overall interpretation of results considering objectives, limitations, multiplicity of analyses, results from similar studies, and other relevant evidence | Paragraphs 3-5 of Discussion |
| Generalisability | 21 | Discuss the generalisability (external validity) of the study results | Paragraph 1 of Discussion |
| Other information | | | |
| Funding | 22 | Give the source of funding and the role of the funders for the present study and, if applicable, for the original study on which the present article is based | Submission information (not in manuscript file as per Journal instructions) |
