# Supplementary material for: Length of Stay After Childbirth in 92 Countries and Associated Factors in 30 Low- and Middle-Income Countries: Compilation of Reported Data and a Cross-sectional Analysis from Nationally Representative Surveys
Source: PLoS Med. 2016 Mar 8;13(3):e1001972. doi: 10.1371/journal.pmed.1001972 (PMC4783077; doi:10.1371/journal.pmed.1001972)
Supplement: S1 Analysis Plan — (DOCX) [file pmed.1001972.s002.docx]

| **Broad overview plan: 9 April 2015** | |
| --- | --- |
| **Plan** | **Decision made/Subsequent changes** |
| 1. Use our 57 country data-set to investigate LOS for the most recent birth by key explanatory variables | **Done** |
| 1. Also look at those getting weighed and getting pre-discharge check (consequence of short LoS) | **Done** Data pulled but not written up for this paper as they are about consequences rather than risk factors. Reason for change: Looking at things that may be the outcome of the short length of stay is another objective. We are planning to do more work in this area to look at determinants to getting a pre-discharge check and of having a birth weighed where we look at many other risk factors for these outcomes. For this future paper, length of stay will be one explanatory variable among others. |
| 1. Key steps will be |  |
| - Identify countries which asked about LoS | **Done** |
| - Identify and operationalize variables: outcome (linear/categorical) & exposures | **Done** |
| - Produce descriptive tables | **Done** |
| - Conduct multivariable analyses (linear and logistic) for DHS data – interactions to be decided a-priori based on conceptualization | **Done** |

| **Draft Analysis Plans April 28- May 5th 2015** | **Decision made/Subsequent changes** |
| --- | --- |
| 1. Operationalize outcome variables | |
| - 1. Find countries with data. Review countries to identify those with length of stay data | **Done.** 31/57 countries have relevant questions (including Moldova which was found later with variable with different name) |
| - 1. How to deal with data measures in hours, days and weeks. Responses “1 week” -- is that 7 days or 10 days? | **Done. Decision:** Use as 10 days (Mid-point (so 1 week is 10 days, 2 weeks is 17 days etc….).  Conversion of days/weeks to hours  Hours: up to 23 reported in hours. If reported in hours, use hours even if > 23 hours (so, if say 48 hours or 36 hours use that. If days,  Days: (i.e., 201 = 1 day) – use (number of day*24)+12 hours, so 1 day is 36 hours, 2 days is (2x24)+12 =  1 week is 10 DAYS: 2 weeks is 17 days, 3 weeks is…. (N of weeks*7)+3.  Do this in order weeks, then days. |
| - 1. Check LOS including 96-99 and whether appropriate to recode as missing | **Done** |
| - 1. Decide outliers’ cut-off and transformation (descriptive and/or analytical). Reviewed LSE paper (Kulinskaya E 2005): 3 alternatives differ in outliers handling - Remove outliers from raw data - Remove outliers from log-transformed data - adaptive model building with predicted v observed | **Decision:** Descriptive analysis: truncate from raw data at three weeks based on small percentage but also sense that > 3 weeks is long; Cut-off for outliers: include those staying 3 weeks, which with recode method above would come out at 588 hours; **Decision:** Regression: truncate outliers from log-transformed data (based on percentiles). **CHANGE and** s**ubsequent decision**: Decided not to use log-transformed data because it was easier to understand untransformed data. Decided to check with a sensitivity analysis and because direction and relative magnitude of the effect did not differ, we decided instead decided to use the same truncation as in the descriptive analyses. |
| - 1. DECIDE whether to show results as linear (number of hours) or logistic (cutoff; too short or (short, okay, too long) | **Decision:** Do both linear and logistic. Logistic to be done separately for vaginal and c-sections as there different definitions of what is too short. Decided to do too short but not too long as we could not justify saying a stay was too long without knowing if there were major complications requiring a longer stay. By contrast, there are standards  Linear for everyone (excluding outliers)   - - separate model for vaginal and C-section deliveries     - - - another model with both modes of deliveries, including as covariate   for the minimum required stay |
| - 1. Present LoS **in** days or hours? | **Decision:** Keep LOS in hours but will present in days in descriptive (hard to understand long periods in hours) and in hours for effect estimates in linear regression |
| - 1. Select cutoff for “too short” (above or below the cutoff – risk of staying less time than cutoff – 24 for vaginal, 72 or 96 hrs.? for C-section) according to literature | **Decision:** Logistic for too short (<24 vaginal/<72 for C-section) |
| 1. Operationalize explanatory variables | |
| Include:   - mother’s age & parity (birth order); - sex of child; - level of delivery facility (hospital or lower); - delivery facility sector (public-private); - Birth attendant; - Residence; region - cesarean section (Mode-of-delivery) (index child); - child survival; (index child); - SES: Wealth quintile, Marital status   Add other relevant variables and operationalize these | **Done. Decision:** Variables to add   1. Add education level to dataset 2. Add birth weight (cutoffs 1999, 2499) into dataset (add 2500-2999, 3000-3499, 3500+). Check birthweight variable, and how missingness handled (recode 9999 into missing). 3. Add multiple births; Twins and how to show sex for discordant. Decision Twins and sex: check if any change in effect in multivariable 4. Add wantedness of child   **CHANGE and subsequent decision:** Dropped two variables we originally thought to includ**e**   1. Take out “world region” variable. This was not a well thought through variable but was included because we had considered if for another set of descriptive papers on use of family planning, antenatal care and delivery care where we were trying to get regional picture as well as country; on reflection we had no reason to really think length of stay would vary by world region and did not want to adjust for region and country 2. Decided to drop level of facility because there were data quality issues whereby some countries did not have response options that distinguished between levels of facilities for private sector (i.e. they had public hospital and public health centre but only private facility as response options); also we saw no effect of level on length of stay for those countries that did have the data.   **Decision:** Check “checked before discharge” variable is in dataset and add to data for analysis. This step is not part of this paper as mentioned on overview |
| Assess percent missing (drop or use imputation) | **Decision:** Missing data confirmed to be negligible except birthweight (20,000 missing values). **Decision:** Check at multivariable stage with/without as sample reduces by 20%. So for multivariable, include one model with birthweight, then run model without and compare. |
| Add additional explanatory variables selected to the data sets and check how operationalized | **Done.**   - 1. Merged child survival variables: Survived, died before/on day of discharge, died after discharge   2. Recode child survival converted to hours and compared to LOS   3. Check estimates for two categories of died (does it make sense?) **Decision**: yes   4. How to check timing of death v discharge: need to recode LOS into days to compare in days   5. Check how length of life was converted by midpoint method from months/weeks to days   6. How to check timing of death v discharge: need to recode LOS into days to compare in days. **Decision:**   7. DAY Zero for death+ <23 hours; day 1 for death= day 1 for discharge=36 hours etc. Day 7-13 of death = week 1 of discharge=10 days; etc. up to day 21-27 of death = week 3 of discharge =day 24 of discharge. If baby dies in months= then 1 month= 28 days+15 days. 2 months =75 days; month 3 =(3*30)+15   8. Look at types of birth attendant by country to understand extent of variability   9. Check women who were delivered by nurse/midwives and had C-section**. Decision:** exclude attendant in model with C-section. Put in discussion/limitations as an issue of misclassification.   10. Check how cesarean handled in our Delivery paper: "Cesarean-section births reported by women who delivered in a home environment were recoded as normal deliveries, regardless of who assisted with the delivery. Cesarean-sections that were reported in facilities, but where the highest level of delivery attendant was reported as general facility staff (e.g., patient attendant or sanitary), husband/friend/relative, other person or no one, were recoded as missing the mode of delivery." |
| 1. Prepare descriptive tables & graphs | |
| 1. Country-level descriptives to be weighted by sample weight 2. Means/medians 3. total sample number (all women surveyed) and % delivering in facility, 4. Add missing LOS data among deliveries in facility 5. Also check % above outlier cutoff 6. Think about how to weight the median 7. Box plots (for countries, total and by mode; for total; and by mode (vaginal singletons/twins, cesarean section) 8. Kaplan-Meier for total; and by mode (vaginal singletons/twins, cesarean section) 9. Check where univariable and multivariable results are consistent or if have flipped effect e.g. wealth (and all others) make sure no errors 10. Check missing and describe how handled 11. double-check numbers add up to same totals for every covariate + missing 12. Check sample size same across all tables 13. Double check countries where mean is much higher than median (Maldives) | 1. **Done.** 2. **Done.** 3. **Done**. **Decision:** To only, show total by country. **Change and subsequent decision:** use violin plots on reviewer’s suggestion as this better shows the distribution of the data 4. **Done.** **Decision:** keep KM curve all and by cesarean, box plots for country. 5. **Done**. Change reference categories to make consistent in descriptive and multivariable: marital status, birth attendant, age group 6. **Done** 7. **Done** 8. **Done** 9. **Done** |
| 1. Multivariable analyses | |
| 1. Linear – total and stratified by mode adjusting always for country 2. Random intercept multilevel model by country 3. Drop determinants not associated at p< 0.05 (unless effect size changes by >10 percent 4. Logistic on proportions too short 5. Pre-specify effect modifiers and only test those; Make conceptual decision on effect modifiers consider examining the following:    - Wealth    - C-section    - Delivery | - 1. **Done. Decision:** Model build-up approach  1. Run set of models each variable with/without C-section to understand results (add column to Table 1) 2. MODEL 1: linear, C-section as covariate 3. MODEL 2: linear, split into 2 datasets; run by mode of delivery 4. MODEL 3: logistic (too short or ok, different cutoffs by mode) on the split dataset, separately by mode 5. MODEL 4: tbd if decide to include effect modifiers    1. **Done**    2. **Done. CHANGE and subsequent decision:** keep all variables in in response to reviewer’s comments because this fit better with our conceptual framework approach    3. **Done**    4. **Decision:** Conceptual decision on effect modification: do stratified analyses by mode of delivery rather than interaction   **Decision taken earlier in step 1:** Run final linear models using log-transformed length of stay outcomes to assess influence of data skewness (sensitivity analysis)   - 1. **Decision take earlier in step 2:** Bangladesh has no birthweight data. Exclude birthweight to see if this influences effect of other variables (sensitivity analysis). |
