# Supplementary material for: Length of Stay After Childbirth in 92 Countries and Associated Factors in 30 Low- and Middle-Income Countries: Compilation of Reported Data and a Cross-sectional Analysis from Nationally Representative Surveys
Source: PLoS Med. 2016 Mar 8;13(3):e1001972. doi: 10.1371/journal.pmed.1001972 (PMC4783077; doi:10.1371/journal.pmed.1001972)
Supplement: S1 Data — (DOCX) [file pmed.1001972.s003.docx]

|  | Country (year) | Source | Access to datasets, reports or tables of indicators | Source of additional OECD data on trends: Access |
| --- | --- | --- | --- | --- |
| 1 | Albania 2009 | DHS | DHS access instructions: (accessed 22/November 2015) http://www.dhsprogram.com/data/Access-Instructions.cfm | Not applicable |
| 2 | Azerbaijan 2006 | DHS | DHS access instructions: (accessed 22/November 2015) http://www.dhsprogram.com/data/Access-Instructions.cfm | Not applicable |
| 3 | Bangladesh 2011 | DHS | DHS access instructions: (accessed 22/November 2015) http://www.dhsprogram.com/data/Access-Instructions.cfm | Not applicable |
| 4 | Benin 2006 | DHS | DHS access instructions: (accessed 22/November 2015) http://www.dhsprogram.com/data/Access-Instructions.cfm | Not applicable |
| 5 | Bolivia 2008 | DHS | DHS access instructions: (accessed 22/November 2015) http://www.dhsprogram.com/data/Access-Instructions.cfm | Not applicable |
| 6 | Dominican Republic 2007 | DHS | DHS access instructions: (accessed 22/November 2015) http://www.dhsprogram.com/data/Access-Instructions.cfm | Not applicable |
| 7 | Egypt 2008 | DHS | DHS access instructions: (accessed 22/November 2015) http://www.dhsprogram.com/data/Access-Instructions.cfm | Not applicable |
| 8 | Gabon 2012 | DHS | DHS access instructions: (accessed 22/November 2015) http://www.dhsprogram.com/data/Access-Instructions.cfm | Not applicable |
| 9 | Ghana 2008 | DHS | DHS access instructions: (accessed 22/November 2015) http://www.dhsprogram.com/data/Access-Instructions.cfm | Not applicable |
| 10 | Guyana 2009 | DHS | DHS access instructions: (accessed 22/November 2015) http://www.dhsprogram.com/data/Access-Instructions.cfm | Not applicable |
| 11 | Haiti 2012 | DHS | DHS access instructions: (accessed 22/November 2015) http://www.dhsprogram.com/data/Access-Instructions.cfm | Not applicable |
| 12 | Honduras 2012 | DHS | DHS access instructions: (accessed 22/November 2015) http://www.dhsprogram.com/data/Access-Instructions.cfm | Not applicable |
| 13 | India 2006 | DHS | DHS access instructions: (accessed 22/November 2015) http://www.dhsprogram.com/data/Access-Instructions.cfm | Not applicable |
| 14 | Jordan 2007 | DHS | DHS access instructions: (accessed 22/November 2015) http://www.dhsprogram.com/data/Access-Instructions.cfm | Not applicable |
| 15 | Kenya 2009 | DHS | DHS access instructions: (accessed 22/November 2015) http://www.dhsprogram.com/data/Access-Instructions.cfm | Not applicable |
| 16 | Lesotho 2009 | DHS | DHS access instructions: (accessed 22/November 2015) http://www.dhsprogram.com/data/Access-Instructions.cfm | Not applicable |
| 17 | Liberia 2007 | DHS | DHS access instructions: (accessed 22/November 2015) http://www.dhsprogram.com/data/Access-Instructions.cfm | Not applicable |
| 18 | Madagascar 2009 | DHS | DHS access instructions: (accessed 22/November 2015) http://www.dhsprogram.com/data/Access-Instructions.cfm | Not applicable |
| 19 | Maldives 2009 | DHS | DHS access instructions: (accessed 22/November 2015) http://www.dhsprogram.com/data/Access-Instructions.cfm | Not applicable |
| 20 | Republic of Moldova 2005 | DHS | DHS access instructions: (accessed 22/November 2015) http://www.dhsprogram.com/data/Access-Instructions.cfm | Not applicable |
| 21 | Namibia 2007 | DHS | DHS access instructions: (accessed 22/November 2015) http://www.dhsprogram.com/data/Access-Instructions.cfm | Not applicable |
| 22 | Nigeria 2008 | DHS | DHS access instructions: (accessed 22/November 2015) http://www.dhsprogram.com/data/Access-Instructions.cfm | Not applicable |
| 23 | Pakistan 2007 | DHS | DHS access instructions: (accessed 22/November 2015) http://www.dhsprogram.com/data/Access-Instructions.cfm | Not applicable |
| 24 | Sao Tome & Principe 2009 | DHS | DHS access instructions: (accessed 22/November 2015) http://www.dhsprogram.com/data/Access-Instructions.cfm | Not applicable |
| 25 | Sierra Leone 2008 | DHS | DHS access instructions: (accessed 22/November 2015) http://www.dhsprogram.com/data/Access-Instructions.cfm | Not applicable |
| 26 | Swaziland 2007 | DHS | DHS access instructions: (accessed 22/November 2015) http://www.dhsprogram.com/data/Access-Instructions.cfm | Not applicable |
| 27 | Timor-Leste 2010 | DHS | DHS access instructions: (accessed 22/November 2015) http://www.dhsprogram.com/data/Access-Instructions.cfm | Not applicable |
| 28 | Uganda 2011 | DHS | DHS access instructions: (accessed 22/November 2015) http://www.dhsprogram.com/data/Access-Instructions.cfm | Not applicable |
| 29 | Ukraine 2007 | DHS | DHS access instructions: (accessed 22/November 2015) http://www.dhsprogram.com/data/Access-Instructions.cfm | Not applicable |
| 30 | Zambia 2007 | DHS | DHS access instructions: (accessed 22/November 2015) http://www.dhsprogram.com/data/Access-Instructions.cfm | Not applicable |
| 31 | Algeria 2012-13 | MICS | MICS Survey Reports. (accessed 22 November 2015) http://mics.unicef.org/surveys | Not applicable |
| 32 | Barbados 2012 | MICS | MICS Survey Reports. (accessed 22 November 2015) http://mics.unicef.org/surveys | Not applicable |
| 33 | Belarus 2012 | MICS | MICS Survey Reports. (accessed 22 November 2015) http://mics.unicef.org/surveys | Not applicable |
| 34 | Belize 2011 | MICS | MICS Survey Reports. (accessed 22 November 2015) http://mics.unicef.org/surveys | Not applicable |
| 35 | Cameroon 2014 | MICS | MICS Survey Reports. (accessed 22 November 2015) http://mics.unicef.org/surveys | Not applicable |
| 36 | Cuba 2014 | MICS | MICS Survey Reports. (accessed 22 November 2015) http://mics.unicef.org/surveys | Not applicable |
| 37 | El Salvador 2014 | MICS | MICS Survey Reports. (accessed 22 November 2015) http://mics.unicef.org/surveys | Not applicable |
| 38 | Guinea-Bissau 2014 | MICS | MICS Survey Reports. (accessed 22 November 2015) http://mics.unicef.org/surveys | Not applicable |
| 39 | Kosovo 2013-14 | MICS | MICS Survey Reports. (accessed 22 November 2015) http://mics.unicef.org/surveys | Not applicable |
| 40 | Kyrgyzstan 2014 | MICS | MICS Survey Reports. (accessed 22 November 2015) http://mics.unicef.org/surveys | Not applicable |
| 41 | Lao 2011-12 | MICS | MICS Survey Reports. (accessed 22 November 2015) http://mics.unicef.org/surveys | Not applicable |
| 42 | Malawi 2013-14 | MICS | MICS Survey Reports. (accessed 22 November 2015) http://mics.unicef.org/surveys | Not applicable |
| 43 | Mongolia 2013-14 | MICS | MICS Survey Reports. (accessed 22 November 2015) http://mics.unicef.org/surveys | Not applicable |
| 44 | Nepal 2014 | MICS | MICS Survey Reports. (accessed 22 November 2015) http://mics.unicef.org/surveys | Not applicable |
| 45 | Palestine 2014 | MICS | MICS Survey Reports. (accessed 22 November 2015) http://mics.unicef.org/surveys | Not applicable |
| 46 | Panama 2013 | MICS | MICS Survey Reports. (accessed 22 November 2015) http://mics.unicef.org/surveys | Not applicable |
| 47 | Qatar 2012 | MICS | MICS Survey Reports. (accessed 22 November 2015) http://mics.unicef.org/surveys | Not applicable |
| 48 | Saint Lucia 2012 | MICS | MICS Survey Reports. (accessed 22 November 2015) http://mics.unicef.org/surveys | Not applicable |
| 49 | Sudan 2014 | MICS | MICS Survey Reports. (accessed 22 November 2015) http://mics.unicef.org/surveys | Not applicable |
| 50 | Viet Nam 2013-14 | MICS | MICS Survey Reports. (accessed 22 November 2015) http://mics.unicef.org/surveys | Not applicable |
| 51 | Zimbabwe 2014 | MICS | MICS Survey Reports. (accessed 22 November 2015) http://mics.unicef.org/surveys | Not applicable |
| 52 | Georgia 2005 | CDC - RHS | RHS- CDC reports http://stacks.cdc.gov/view/cdc/8275 | Not applicable |
| 53 | Australia 2011 | OECD 2006-13 | LoS (accessed 22 November 2015 http://www.oecd-ilibrary.org/social-issues-migration-health/average-length-of-stay-childbirth-2014-1_l-o-s-childbirth-table-2014-1-en | Not available |
| 54 | Austria 2012 | OECD 2006-13 | LoS (accessed 22 November 2015 http://www.oecd-ilibrary.org/social-issues-migration-health/average-length-of-stay-childbirth-2014-1_l-o-s-childbirth-table-2014-1-en | OECD (2015), Length of hospital stay. doi: 10.1787/8dda6b7a-en (Accessed on 13 August 2015) https://data.oecd.org/healthcare/length-of-hospital-stay.htm#indicator-chart |
| 55 | Belgium 2011 | OECD 2006-13 | LoS (accessed 22 November 2015 http://www.oecd-ilibrary.org/social-issues-migration-health/average-length-of-stay-childbirth-2014-1_l-o-s-childbirth-table-2014-1-en | OECD (2015), Length of hospital stay. doi: 10.1787/8dda6b7a-en (Accessed on 13 August 2015) https://data.oecd.org/healthcare/length-of-hospital-stay.htm#indicator-chart |
| 56 | Canada 2011 | OECD 2006-13 | LoS (accessed 22 November 2015 http://www.oecd-ilibrary.org/social-issues-migration-health/average-length-of-stay-childbirth-2014-1_l-o-s-childbirth-table-2014-1-en | OECD (2015), Length of hospital stay. doi: 10.1787/8dda6b7a-en (Accessed on 13 August 2015) https://data.oecd.org/healthcare/length-of-hospital-stay.htm#indicator-chart |
| 57 | Chile 2011 | OECD 2006-13 | LoS (accessed 22 November 2015 http://www.oecd-ilibrary.org/social-issues-migration-health/average-length-of-stay-childbirth-2014-1_l-o-s-childbirth-table-2014-1-en | Not available |
| 58 | Czech Republic 2012 | OECD 2006-13 | LoS (accessed 22 November 2015 http://www.oecd-ilibrary.org/social-issues-migration-health/average-length-of-stay-childbirth-2014-1_l-o-s-childbirth-table-2014-1-en | OECD (2015), Length of hospital stay. doi: 10.1787/8dda6b7a-en (Accessed on 13 August 2015) https://data.oecd.org/healthcare/length-of-hospital-stay.htm#indicator-chart |
| 59 | Denmark 2010 | OECD 2006-13 | LoS (accessed 22 November 2015 http://www.oecd-ilibrary.org/social-issues-migration-health/average-length-of-stay-childbirth-2014-1_l-o-s-childbirth-table-2014-1-en | OECD (2015), Length of hospital stay. doi: 10.1787/8dda6b7a-en (Accessed on 13 August 2015) https://data.oecd.org/healthcare/length-of-hospital-stay.htm#indicator-chart |
| 60 | Finland 2012 | OECD 2006-13 | LoS (accessed 22 November 2015 http://www.oecd-ilibrary.org/social-issues-migration-health/average-length-of-stay-childbirth-2014-1_l-o-s-childbirth-table-2014-1-en | OECD (2015), Length of hospital stay. doi: 10.1787/8dda6b7a-en (Accessed on 13 August 2015) https://data.oecd.org/healthcare/length-of-hospital-stay.htm#indicator-chart |
| 61 | France 2012 | OECD 2006-13 | LoS (accessed 22 November 2015 http://www.oecd-ilibrary.org/social-issues-migration-health/average-length-of-stay-childbirth-2014-1_l-o-s-childbirth-table-2014-1-en | OECD (2015), Length of hospital stay. doi: 10.1787/8dda6b7a-en (Accessed on 13 August 2015) https://data.oecd.org/healthcare/length-of-hospital-stay.htm#indicator-chart |
| 62 | Germany 2012 | OECD 2006-13 | LoS (accessed 22 November 2015 http://www.oecd-ilibrary.org/social-issues-migration-health/average-length-of-stay-childbirth-2014-1_l-o-s-childbirth-table-2014-1-en | Not available |
| 63 | Greece 2007 | OECD 2006-13 | LoS (accessed 22 November 2015 http://www.oecd-ilibrary.org/social-issues-migration-health/average-length-of-stay-childbirth-2014-1_l-o-s-childbirth-table-2014-1-en | Not available |
| 64 | Hungary 2012 | OECD 2006-13 | LoS (accessed 22 November 2015 http://www.oecd-ilibrary.org/social-issues-migration-health/average-length-of-stay-childbirth-2014-1_l-o-s-childbirth-table-2014-1-en | OECD (2015), Length of hospital stay. doi: 10.1787/8dda6b7a-en (Accessed on 13 August 2015) https://data.oecd.org/healthcare/length-of-hospital-stay.htm#indicator-chart |
| 65 | Iceland 2009 | OECD 2006-13 | LoS (accessed 22 November 2015 http://www.oecd-ilibrary.org/social-issues-migration-health/average-length-of-stay-childbirth-2014-1_l-o-s-childbirth-table-2014-1-en | Not available |
| 66 | Ireland 2012 | OECD 2006-13 | LoS (accessed 22 November 2015 http://www.oecd-ilibrary.org/social-issues-migration-health/average-length-of-stay-childbirth-2014-1_l-o-s-childbirth-table-2014-1-en | OECD (2015), Length of hospital stay. doi: 10.1787/8dda6b7a-en (Accessed on 13 August 2015) https://data.oecd.org/healthcare/length-of-hospital-stay.htm#indicator-chart |
| 67 | Israel 2012 | OECD 2006-13 | LoS (accessed 22 November 2015 http://www.oecd-ilibrary.org/social-issues-migration-health/average-length-of-stay-childbirth-2014-1_l-o-s-childbirth-table-2014-1-en | OECD (2015), Length of hospital stay. doi: 10.1787/8dda6b7a-en (Accessed on 13 August 2015) https://data.oecd.org/healthcare/length-of-hospital-stay.htm#indicator-chart |
| 68 | Italy 2012 | OECD 2006-13 | LoS (accessed 22 November 2015 http://www.oecd-ilibrary.org/social-issues-migration-health/average-length-of-stay-childbirth-2014-1_l-o-s-childbirth-table-2014-1-en | OECD (2015), Length of hospital stay. doi: 10.1787/8dda6b7a-en (Accessed on 13 August 2015) https://data.oecd.org/healthcare/length-of-hospital-stay.htm#indicator-chart |
| 69 | Luxembourg 2012 | OECD 2006-13 | LoS (accessed 22 November 2015 http://www.oecd-ilibrary.org/social-issues-migration-health/average-length-of-stay-childbirth-2014-1_l-o-s-childbirth-table-2014-1-en | OECD (2015), Length of hospital stay. doi: 10.1787/8dda6b7a-en (Accessed on 13 August 2015) https://data.oecd.org/healthcare/length-of-hospital-stay.htm#indicator-chart |
| 70 | Mexico 2012 | OECD 2006-13 | LoS (accessed 22 November 2015 http://www.oecd-ilibrary.org/social-issues-migration-health/average-length-of-stay-childbirth-2014-1_l-o-s-childbirth-table-2014-1-en | Not available |
| 71 | Netherlands 2012 | OECD 2006-13 | LoS (accessed 22 November 2015 http://www.oecd-ilibrary.org/social-issues-migration-health/average-length-of-stay-childbirth-2014-1_l-o-s-childbirth-table-2014-1-en | OECD (2015), Length of hospital stay. doi: 10.1787/8dda6b7a-en (Accessed on 13 August 2015) https://data.oecd.org/healthcare/length-of-hospital-stay.htm#indicator-chart |
| 72 | New Zealand 2012 | OECD 2006-13 | LoS (accessed 22 November 2015 http://www.oecd-ilibrary.org/social-issues-migration-health/average-length-of-stay-childbirth-2014-1_l-o-s-childbirth-table-2014-1-en | OECD (2015), Length of hospital stay. doi: 10.1787/8dda6b7a-en (Accessed on 13 August 2015) https://data.oecd.org/healthcare/length-of-hospital-stay.htm#indicator-chart |
| 73 | Norway 2010 | OECD 2006-13 | LoS (accessed 22 November 2015 http://www.oecd-ilibrary.org/social-issues-migration-health/average-length-of-stay-childbirth-2014-1_l-o-s-childbirth-table-2014-1-en | OECD (2015), Length of hospital stay. doi: 10.1787/8dda6b7a-en (Accessed on 13 August 2015) https://data.oecd.org/healthcare/length-of-hospital-stay.htm#indicator-chart |
| 74 | Poland 2012 | OECD 2006-13 | LoS (accessed 22 November 2015 http://www.oecd-ilibrary.org/social-issues-migration-health/average-length-of-stay-childbirth-2014-1_l-o-s-childbirth-table-2014-1-en | OECD (2015), Length of hospital stay. doi: 10.1787/8dda6b7a-en (Accessed on 13 August 2015) https://data.oecd.org/healthcare/length-of-hospital-stay.htm#indicator-chart |
| 75 | Portugal 2009 | OECD 2006-13 | LoS (accessed 22 November 2015 http://www.oecd-ilibrary.org/social-issues-migration-health/average-length-of-stay-childbirth-2014-1_l-o-s-childbirth-table-2014-1-en | Not available |
| 76 | Slovakia 2012 | OECD 2006-13 | LoS (accessed 22 November 2015 http://www.oecd-ilibrary.org/social-issues-migration-health/average-length-of-stay-childbirth-2014-1_l-o-s-childbirth-table-2014-1-en | Not available |
| 77 | Slovenia 2012 | OECD 2006-13 | LoS (accessed 22 November 2015 http://www.oecd-ilibrary.org/social-issues-migration-health/average-length-of-stay-childbirth-2014-1_l-o-s-childbirth-table-2014-1-en | OECD (2015), Length of hospital stay. doi: 10.1787/8dda6b7a-en (Accessed on 13 August 2015) https://data.oecd.org/healthcare/length-of-hospital-stay.htm#indicator-chart |
| 78 | Republic of Korea 2013 | OECD 2006-13 | LoS (accessed 22 November 2015 http://www.oecd-ilibrary.org/social-issues-migration-health/average-length-of-stay-childbirth-2014-1_l-o-s-childbirth-table-2014-1-en | OECD (2015), Length of hospital stay. doi: 10.1787/8dda6b7a-en (Accessed on 13 August 2015) https://data.oecd.org/healthcare/length-of-hospital-stay.htm#indicator-chart |
| 79 | Spain 2012 | OECD 2006-13 | LoS (accessed 22 November 2015 http://www.oecd-ilibrary.org/social-issues-migration-health/average-length-of-stay-childbirth-2014-1_l-o-s-childbirth-table-2014-1-en | OECD (2015), Length of hospital stay. doi: 10.1787/8dda6b7a-en (Accessed on 13 August 2015) https://data.oecd.org/healthcare/length-of-hospital-stay.htm#indicator-chart |
| 80 | Sweden 2010 | OECD 2006-13 | LoS (accessed 22 November 2015 http://www.oecd-ilibrary.org/social-issues-migration-health/average-length-of-stay-childbirth-2014-1_l-o-s-childbirth-table-2014-1-en | OECD (2015), Length of hospital stay. doi: 10.1787/8dda6b7a-en (Accessed on 13 August 2015) https://data.oecd.org/healthcare/length-of-hospital-stay.htm#indicator-chart |
| 81 | Switzerland 2012 | OECD 2006-13 | LoS (accessed 22 November 2015 http://www.oecd-ilibrary.org/social-issues-migration-health/average-length-of-stay-childbirth-2014-1_l-o-s-childbirth-table-2014-1-en | OECD (2015), Length of hospital stay. doi: 10.1787/8dda6b7a-en (Accessed on 13 August 2015) https://data.oecd.org/healthcare/length-of-hospital-stay.htm#indicator-chart |
| 82 | Turkey 2012 | OECD 2006-13 | LoS (accessed 22 November 2015 http://www.oecd-ilibrary.org/social-issues-migration-health/average-length-of-stay-childbirth-2014-1_l-o-s-childbirth-table-2014-1-en | OECD (2015), Length of hospital stay. doi: 10.1787/8dda6b7a-en (Accessed on 13 August 2015) https://data.oecd.org/healthcare/length-of-hospital-stay.htm#indicator-chart |
| 83 | United Kingdom 2011 | OECD 2006-13 | LoS (accessed 22 November 2015 http://www.oecd-ilibrary.org/social-issues-migration-health/average-length-of-stay-childbirth-2014-1_l-o-s-childbirth-table-2014-1-en | OECD (2015), Length of hospital stay. doi: 10.1787/8dda6b7a-en (Accessed on 13 August 2015) https://data.oecd.org/healthcare/length-of-hospital-stay.htm#indicator-chart |
| 84 | United States 2010 | OECD 2006-13 | LoS (accessed 22 November 2015 http://www.oecd-ilibrary.org/social-issues-migration-health/average-length-of-stay-childbirth-2014-1_l-o-s-childbirth-table-2014-1-en | Not available |
| 85 | Bulgaria 2010 | OECD 2010 or nearest | LoS in 2010 or most recent year (accessed 22 November 2015) http://dx.doi.org/10.1787/888932704494 | Not available |
| 86 | Croatia 2008 | OECD 2010 or nearest | LoS in 2010 or most recent year (accessed 22 November 2015) http://dx.doi.org/10.1787/888932704494 | Not available |
| 87 | Cyprus 2010 | OECD 2010 or nearest | LoS in 2010 or most recent year (accessed 22 November 2015) http://dx.doi.org/10.1787/888932704494 | Not available |
| 88 | FYR of Macedonia 2007 | OECD 2010 or nearest | LoS in 2010 or most recent year (accessed 22 November 2015) http://dx.doi.org/10.1787/888932704494 | Not available |
| 89 | Latvia 2010 | OECD 2010 or nearest | LoS in 2010 or most recent year (accessed 22 November 2015) http://dx.doi.org/10.1787/888932704494 | Not available |
| 90 | Lithuania 2010 | OECD 2010 or nearest | LoS in 2010 or most recent year (accessed 22 November 2015) http://dx.doi.org/10.1787/888932704494 | Not available |
| 91 | Malta 2010 | OECD 2010 or nearest | LoS in 2010 or most recent year (accessed 22 November 2015) http://dx.doi.org/10.1787/888932704494 | Not available |
| 92 | Romania 2010 | OECD 2010 or nearest | LoS in 2010 or most recent year (accessed 22 November 2015) http://dx.doi.org/10.1787/888932704494 | Not available |
