# Supplementary figures and images for: Length of Stay After Childbirth in 92 Countries and Associated Factors in 30 Low- and Middle-Income Countries: Compilation of Reported Data and a Cross-sectional Analysis from Nationally Representative Surveys
Source: PLoS Med. 2016 Mar 8;13(3):e1001972. doi: 10.1371/journal.pmed.1001972 (PMC4783077; doi:10.1371/journal.pmed.1001972)

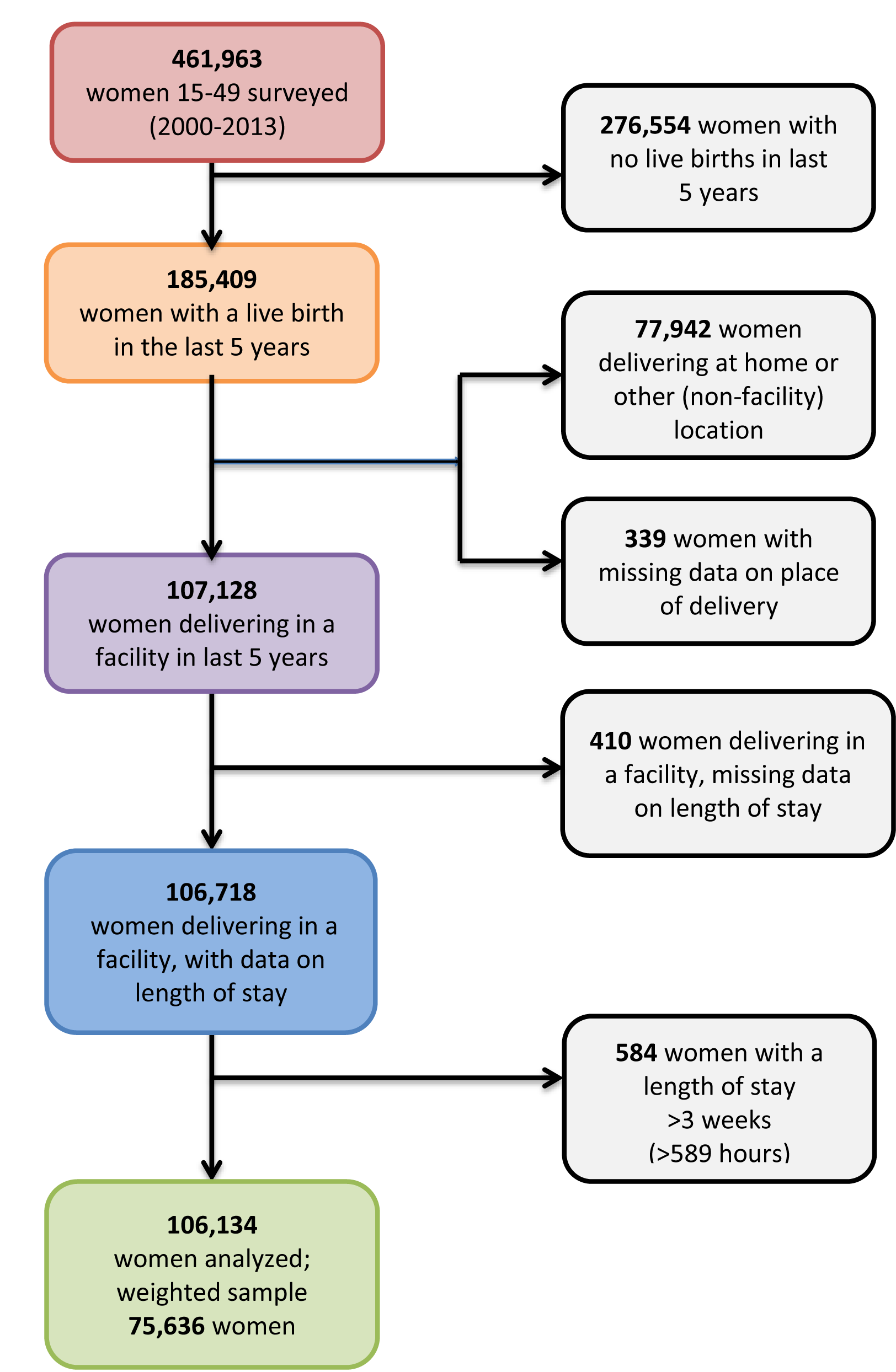

Supplement: S1 Fig — (TIF) [file pmed.1001972.s004.tif]

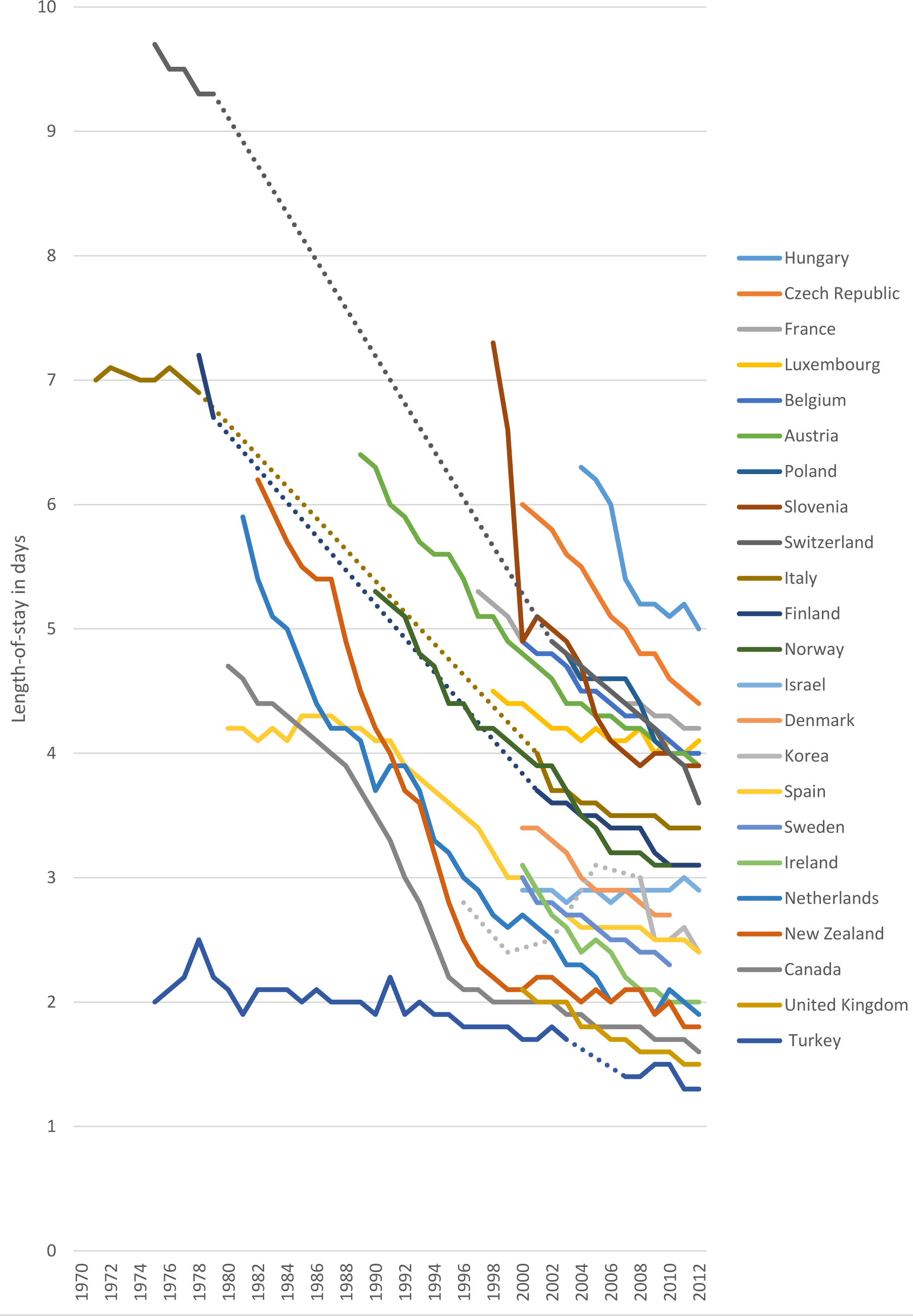

Supplement: S2 Fig — (TIF) [file pmed.1001972.s005.tif]

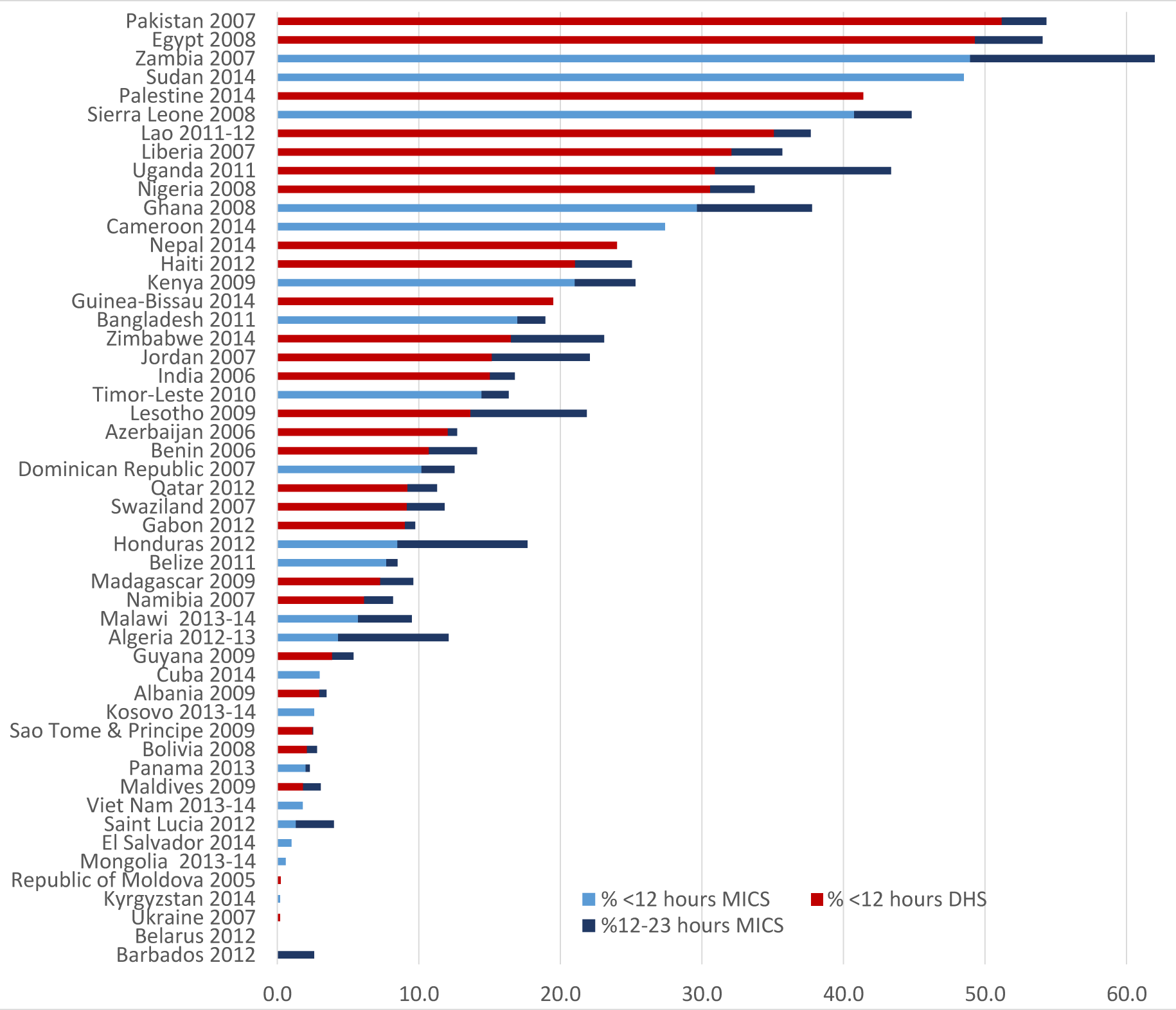

Supplement: S3 Fig — Not all MICS report the percentage with length of stay 12–23 h. MICS include births up to 2 y before the survey; the DHS includes births up to 5 y before the survey. (TIF) [file pmed.1001972.s006.tif]

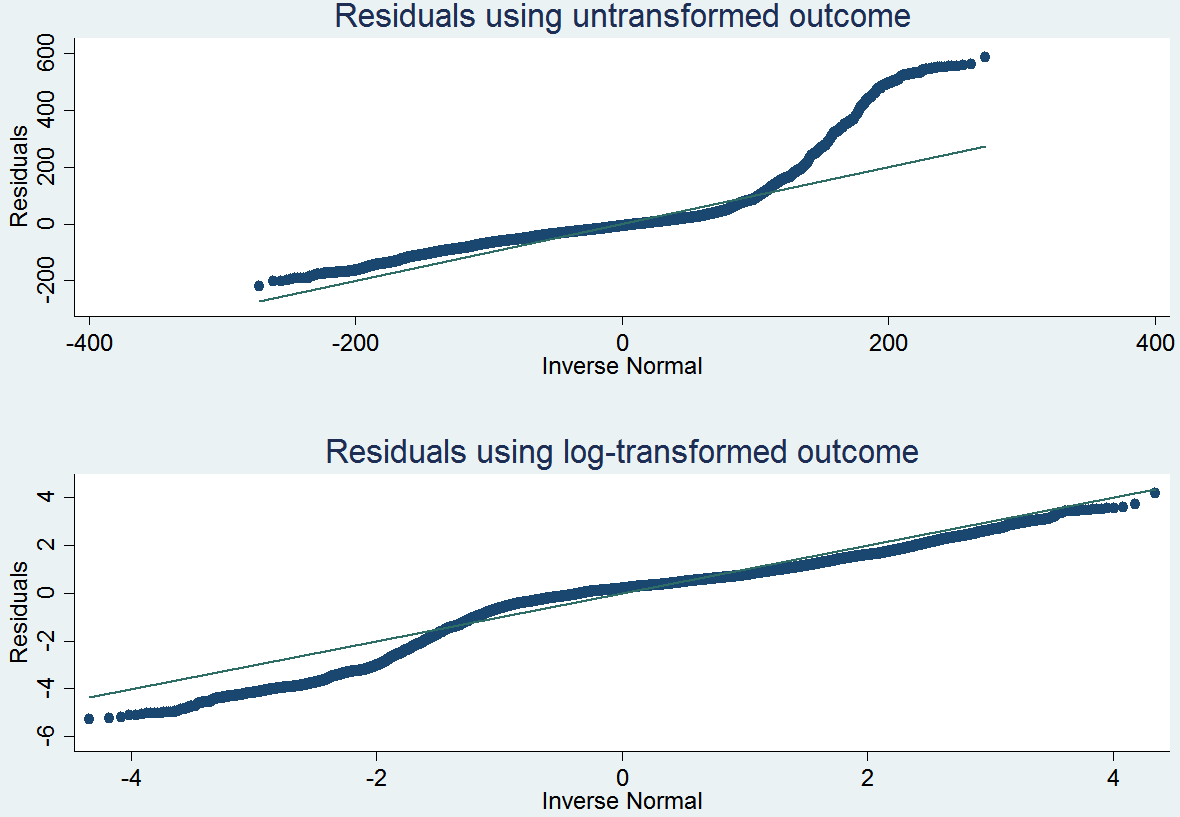

Supplement: S4 Fig — (TIF) [file pmed.1001972.s007.tif]
