# Supplementary material for: Length of Stay After Childbirth in 92 Countries and Associated Factors in 30 Low- and Middle-Income Countries: Compilation of Reported Data and a Cross-sectional Analysis from Nationally Representative Surveys
Source: PLoS Med. 2016 Mar 8;13(3):e1001972. doi: 10.1371/journal.pmed.1001972 (PMC4783077; doi:10.1371/journal.pmed.1001972)
Supplement: S1 Table — Number of women respondents per country, number and percentage (weighted using within-country sample weights) with a live birth in the 5 y before the survey, with at least one birth in a facility, and with a missing length of stay (LoS). Mean length of stay ± standard deviation (SD), in days; median length of stay (interquartile range); percentage of vaginal deliveries staying <24 h; percentage cesarean-section deliveries staying <72 h. (DOCX) [file pmed.1001972.s008.docx]

**S1Table. Number of women respondents per country, number and percentage (weighted using within country sample weights) with a live birth in the 5 years before the survey, with at least one birth in a facility, and with a missing length of stay (LoS). Mean LoS ± standard deviation (SD), in days; median LoS & interquartile range (IQR); percentage vaginal births staying <24 hours; percentage cesarean-section deliveries staying <72 hours.**

| **Region** | **Country (survey year)** | **column a**  **Total women respondents in survey** | **column b**  **Women with live birth within 5 years of survey (% col. b/a)** | **column c**  **Women in col. b with 1+ births in a facility (% col. c/b)** | **column d**  **Women in col. c, missing LoS (% col. d/c)** | **column e**  **Women in col. c with LoS >588 hours (% col. e/c)** | **column f**  **Women in final analysis sample (col. c-(d+e))** | **column g**  **Mean LoS in days (SD)** | **column h**  **Median LoS in days (IQR)** | **column I % vaginal births <24 hours** | **column j**  **% cesarean deliveries <72 hours** |
| --- | --- | --- | --- | --- | --- | --- | --- | --- | --- | --- | --- |
|  | | | | | | | | | | | |
| **Sub-Saharan Africa** | Benin 2006 | 17,794 | 10,521(59.1%) | 8416 (80.0%) | 72 (0.9%) | 73 (0.9%) | 8271 | 3.0 (3.0) | 2.5 (1.5,3.5) | 14.6% | 15.6% |
|  | Gabon 2012 | 8,422 | 3702 (44.0%) | 3427 (92.6%) | 34 (1.0%) | 18 (0.5%) | 3375 | 3.3 (3.1) | 2.5 (1.5,3.5) | 10.6% | 21.5% |
|  | Ghana 2008 | 4,916 | 2099 (42.7%) | 1263 (60.2%) | 7 (0.6%) | 9 (0.7%) | 1247 | 2.5 (3.8) | 1.5 (0.4,2.5) | 41.7% | 16.3% |
|  | Kenya 2008-9 | 8,444 | 3973 (47.1%) | 1858 (46.8%) | 4 (0.2%) | 29 (1.6%) | 1824 | 2.7 (3.5) | 1.5 (0.7,2.5) | 28.2% | 25.8% |
|  | Lesotho 2009 | 7,624 | 2984 (39.1%) | 1822 (61.1%) | 2 (0.1%) | 20 (1.1%) | 1800 | 2.8 (3.7) | 1.5 (1.5,2.5) | 24.2% | 19.3% |
|  | Liberia 2007 | 7,092 | 3928 (55.4%) | 1635 (41.6%) | 16 (1.0%) | 19 (1.1%) | 1601 | 2.0 (3.0) | 1.5 (0.3,2.5) | 37.7% | 45.7% |
|  | Madagascar 2008-9 | 17,375 | 8662 (49.9%) | 3256 (37.6%) | 8 (0.3%) | 8 (0.3%) | 3239 | 3.1 (2.2) | 3.5 (2.5,3.5) | 9.9% | 12.7% |
|  | Namibia 2006-7 | 9,804 | 3898 (39.8%) | 3207 (82.3%) | 7 (0.2%) | 49 (1.5%) | 3151 | 3.3 (3.7) | 2.5 (1.5,3.5) | 9.3% | 26.3% |
|  | Nigeria 2008 | 33,385 | 17,635 (52.8%) | 6431 (36.5%) | 65 (1.0%) | 31 (0.5%) | 6335 | 2.2 (2.9) | 1.5 (0.3,2.5) | 35.0% | 27.1% |
|  | Sao Tome & Principe 2008-9 | 2,615 | 1386 (53.0%) | 1109 (80.0%) | 5 (0.5%) | 6 (0.6%) | 1098 | 4.2 (3.0) | 3.5 (3.5,3.5) | 2.7% | 5.6% |
|  | Sierra Leone 2008 | 7,374 | 4103 (55.6%) | 1061 (25.9%) | 14 (1.3%) | 10 (0.9%) | 1038 | 2.1 (3.3) | 1.5 (0.2,2.5) | 47.2% | 41.7% |
|  | Swaziland 2006-7 | 4,987 | 2134 (42.8%) | 1613 (75.6%) | 2 (0.1%) | 19 (1.2%) | 1592 | 2.8 (3.5) | 1.5 (1.5,2.5) | 13.3% | 13.0% |
|  | Uganda 2011 | 8,674 | 4968 (57.3%) | 2985 (60.1%) | 16 (0.5%) | 23 (0.8%) | 2946 | 1.9 (2.9) | 1.5 (0.3,1.5) | 46.8% | 33.9% |
|  | Zambia 2007 | 7,146 | 4136 (57.9%) | 2090 (50.5%) | 4 (0.2%) | 16 (0.8%) | 2071 | 1.5 (2.8) | 0.5 (0.3,1.5) | 65.0% | 35.6% |
|  | | | | | | | | | | | |
| **North Africa/ Western Asia/ Europe** | Albania 2008-9 | 7,584 | 1310 (17.3%) | 1269 (96.9%) | 2 (0.2%) | 9 (0.7%) | 1259 | 3.6 (3.0) | 2.5 (1.5,3.5) | 4.1% | 10.3% |
|  | Azerbaijan 2006 | 8,444 | 1686 (20.0%) | 1332 (79.0%) | 1 (0.1%) | 1 (0.1%) | 1329 | 5.1 (3.7) | 4.5 (1.5,7.5) | 13.4% | 8.9% |
|  | Egypt 2008* | 16,527 | 7896 (47.8%) | 5714 (72.4%) | 14 (0.2%) | 3 (0.0%) | 5697 | 1.3 (1.7) | 0.5 (0.1,2.5) | 83.2% | 75.3% |
|  | Jordan 2007* | 10,876 | 6446 (59.3%) | 6372 (98.9%) | 4 (0.1%) | 2 (0.0%) | 6365 | 1.9 (1.8) | 1.5 (1.5,2.5) | 26.8% | 33.0% |
|  | Republic of Moldova 2005 | 7,440 | 1387 (18.6%) | 1371 (98.8%) | 1 (0.1%) | 17 (1.3%) | 1353 | 6.2 (3.0) | 5.5 (4.5,6.5) | 0.3% | 2.0% |
|  | Ukraine 2007 | 6,841 | 1072 (15.7%) | 1063 (99.2%) | 3 (0.3%) | 10 (1.0%) | 1050 | 6.6 (3.0) | 5.5 (4.5,7.5) | 0.1% | 1.0% |
|  | | | | | | | | | | | |
| **South/**  **South-East Asia** | Bangladesh 2011* | 17,749 | 7350 (41.4%) | 1934 (26.3%) | 17 (0.9%) | 10 (0.5%) | 1908 | 4.6 (3.6) | 4.5 (1.5,7.5) | 42.4% | 3.3% |
|  | India 2005-6* | 124,385 | 39,677 (31.9%) | 16,457 (41.5%) | 18 (0.1%) | 71 (0.4%) | 16,369 | 4.3 (4.0) | 3.5 (1.5,6.5) | 21.2% | 9.0% |
|  | Maldives 2009* | 7,131 | 3190 (44.7%) | 3060 (95.9%) | 16 (0.5%) | 46 (1.5%) | 2998 | 5.2 (4.2) | 3.5 (2.5,7.5) | 4.3% | 4.1% |
|  | Pakistan 2006-7* | 10,023 | 5677 (56.6%) | 2101 (37.0%) | 3 (0.2%) | 2 (0.1%) | 2095 | 2.0 (3.1) | 0.4 (0.1,2.5) | 69.6% | 9.3% |
|  | Timor-Leste 2009-10 | 13,137 | 6015 (45.8%) | 1512 (25.1%) | 2 (0.1%) | 9 (0.6%) | 1501 | 2.3 (3.0) | 1.5 (1.5,2.5) | 17.3% | 32.7% |
|  | | | | | | | | | | | |
| **Latin America & Caribbean** | Bolivia 2008 | 16,939 | 6472 (38.2%) | 4638 (71.7%) | 1 (0.0%) | 13 (0.3%) | 4625 | 3.1 (2.2) | 2.5 (1.5,3.5) | 3.6% | 25.2% |
|  | Dominican Republic 2007 | 27,195 | 8203 (30.2%) | 8036 (98.0%) | 23 (0.3%) | 11 (0.1%) | 8002 | 2.6 (2.0) | 2.5 (1.5,3.5) | 19.7% | 27.7% |
|  | Guyana 2009 | 4,996 | 1425 (28.5%) | 1302 (91.3%) | 8 (0.6%) | 15 (1.1%) | 1279 | 3.3 (3.0) | 2.5 (1.5,3.5) | 6.0% | 22.3% |
|  | Haiti 2012 | 14,287 | 5218 (36.5%) | 2062 (39.5%) | 21 (1.0%) | 14 (0.7%) | 2027 | 2.6 (3.0) | 1.5 (0.9,3.5) | 29.3% | 16.7% |
|  | Honduras 2011-12 | 22,757 | 8269 (36.3%) | 7011 (84.8%) | 1 (0.0%) | 5 (0.1%) | 7005 | 2.3 (1.9) | 1.5 (1.5,2.5) | 22.5% | 26.1% |
| * Sampled ever-married women aged 15-49 instead of all women 15-49 | | | | | | | | | | | |
