# Supplementary material for: Length of Stay After Childbirth in 92 Countries and Associated Factors in 30 Low- and Middle-Income Countries: Compilation of Reported Data and a Cross-sectional Analysis from Nationally Representative Surveys
Source: PLoS Med. 2016 Mar 8;13(3):e1001972. doi: 10.1371/journal.pmed.1001972 (PMC4783077; doi:10.1371/journal.pmed.1001972)
Supplement: S2 Table — (DOCX) [file pmed.1001972.s009.docx]

**S2 Table. Heatmap showing direction of effects across all models.**

**Vaginal birth**

**length of stay in**

**hours**

**Cesarean**

**birth length of**

**stay in hours**

**Determinant**

**Category**

**Median (IQR), all**

**deliveries**

**Mean**

**+**

**SD, all**

**deliveries**

**Linear**

**regression**

**adjusting for**

**country**

**Linear**

**regression**

**adjusting for**

**country & C-**

**section**

**Linear regression**

**adjusting for all**

**variables**

**Linear regression**

**adjusting for all**

**variables**

**Linear regression**

**adjusting for all**

**variables**

**% Vaginal**

**deliveries too**

**short**

**Logistic**

**regression**

**(vaginal)**

**adjusting for**

**country**

**Logistic regression**

**(vaginal) adjusting**

**for all**

**% cesarean**

**deliveries too**

**short**

**Logistic**

**regression (c-**

**section) adjusting**

**for country**

**Logistic regression (c-**

**section) adjusting for**

**all**

Singleton

2.5 (1.5,3.5)

80.7 ± 88.2

*reference*

*reference*

*reference*

*reference*

*Reference*

33.7

*reference*

*reference*

19.7

*reference*

*reference*

Twins or Triplets

3.5 (1.5,5.5)

114.0 ± 112.7

42.8 (39.3; 46.2)

29.1 (26.0; 32.1)

22.3 (18.8; 25.9)

24.5 (20.6; 28.3)

23.5 (15.7; 31.3)

28.3

0.55 (0.47; 0.64)

0.59 (0.48; 0.72)

17.1

0.51 (0.41; 0.65)

0.57 (0.44; 0.75)

<1,999 grams

3.5 (1.5,6.5)

115.2 ± 115.6

44.4 (41.1; 47.0)

38.4 (36.8; 41.1)

34.3 (31.6; 36.9)

38.8 (36.0; 41.6)

28.6 (22.2; 35.0)

21

0.82 (0.71; 0.93)

0.86 (0.75; 0.99)

18.4

0.59 (0.48; 0.73)

0.69 (0.54; 0.83)

2,000-2,499 grams

2.5 (1.5,3.5)

96.0 ± 87.8

8.8 (7.1; 10.5)

8.2 (6.6; 9.7)

6.9 (5.3; 8.4)

8.0 (6.4; 9.5)

6.6 (2.2; 11.0)

19.5

0.86 (0.80; 0.93)

0.88 (0.81; 0.95)

14.8

0.77 (0.66 0.88)

0.81 (0.70; 0.94)

2,500+ grams

2.5 (1.5,3.5)

89.8 ± 88.6

*reference*

*reference*

*reference*

*reference*

*Reference*

24

*reference*

*reference*

19.4

*reference*

*reference*

Died before/on day of discharge

3.5 (1.5,5.5)

111.7 ± 107.1

41.5 (37.3; 45.8)

32.1 (28.3; 35.9)

25.3 (19.8; 30.9)

22.3 (16.4; 28.1)

29.2 (15.9; 42.4)

23.4

0.54 (0.44; 0.65)

0.54 (0.39; 0.75)

9.8

0.43 (0.30; 0.61)

0.33 (0.19 ; 0.59)

Survived

2.5 (1.5,3.5)

81.0 ± 88.3

*reference*

*reference*

*reference*

*Reference*

*Reference*

33.5

*reference*

*reference*

19.8

*reference*

*reference*

Died after discharge

1.5 (0.7,3.5)

68.8 ± 92.9

1.6 (-1.5; 4.7)

2.2 (-0.6; 4.9)

0.4 (-2.9; 3.8)

1.5 (- 1.7; 4.8)

-6.7 (-17.7; 4.2)

44.5

1.11 (0.99; 1.24)

0.98 (0.84; 1.13)

22.6

0.87 (0.65; 1.15)

0.96 (0.69; 1.35)

Nurse-midwife

1.5 (0.8,2.5)

49.6 ± 64.9

*reference*

*reference*

*reference*

*Reference*

38.2

*reference*

*reference*

Doctor

2.5 (1.5,4.5)

93.7 ± 93.6

43.0 (41.7; 44.3)

21.0 (19.8; 22.2)

19.8 (18.5; 21.2)

8.7 (7.4; 10.0)

30.9

0.60 (0.57; 0.63)

0.64 (0.61; 0.69)

Auxiliary staff/other

1.5 (0.3,2.5)

41.4 ± 61.5

-3.1 (-0.1.; -6.1)

-3.0 (-5.7; -0.3)

-0.9 (-4.4; 2.6)

-0.8 (-4.0; 2.4)

47

1.29 (1.17; 1.42)

1.22 (1.07; 1.41)

Public

2.5 (1.5,3.5)

77.5 ± 88.5

*reference*

*reference*

*reference*

*reference*

*Reference*

31.4

*reference*

*reference*

19.9

*reference*

*reference*

Private

2.5 (1.5,3.5)

85.2 ± 89.0

6.6 (5.5; 7.7)

-3.6 (-4.6; -2.6)

-4.3 (-5.5; -3.1)

1.4 (0.2; 2.7)

-14.1 (-17.1; -11.1)

36.4

0.96 (0.92; 1.00)

1.02 (0.97; 1.08)

19.4

1.58 (1.47; 1.72)

1.51 (1.37; 1.65)

15-19

1.5 (1.5,3.5)

74.2 ± 83.5

-0.2 (-2.1; 1.6)

1.7 (0.0; 3.4)

-0.4 (-2.3; 1.5)

-0.9 (-2.7; 1.0)

0.9 (-5.2; 6.9)

35.7

1.00 (0.93; 1.08)

1.00 (0.92; 1.10)

17.9

0.94 (0.80; 1.10)

1.00 (0.83; 1.19)

20-24

2.5 (1.5,3.5)

81.4 ± 86.0

*reference*

*reference*

*reference*

*reference*

*Reference*

30.9

*reference*

*reference*

19.1

*reference*

*reference*

25-29

2.5 (1.5,3.5)

84.2 ± 90.4

2.4 (1.2; 3.7)

0.7 (-0.4; 1.7)

2.5 (1.2; 3.7)

2.7 (1.4; 3.9)

1.3 (-2.3; 4.9)

32.9

0.97 (0.93; 1.02)

0.96 (0.91; 1.02)

19.4

1.01 (0.92; 1.12)

0.99 (0.88; 1.11)

30-34

2.5 (1.5,3.5)

83.5 ± 91.0

6.4 (5.0; 7.7)

2.2 (1.0; 3.4)

4.9 (3.4; 6.3)

4.0 (2.5; 5.4)

8.1 (4.0; 12.2)

34.5

0.95 (0.90; 1.00)

0.91 (0.84; 0.97)

20.1

0.87 (0.78; 0.96)

0.81 (0.71; 0.92)

35-39

2.5 (1.5,3.5)

76.7 ± 90.9

9.0 (7.5; 10.6)

4.2 ( 2.8; 5.6)

7.5 (5.7; 9.3)

6.2 (4.4; 8.0)

14.0 (9.1; 19.0)

38.3

0.92 (0.86; 0.98)

0.86 (0.79; 0.94)

20.7

0.82 (0.72; 0.93)

0.76 (0.65; 0.89)

40-44

2.5 (1.5,3.5)

70.1 ± 87.0

12.5 (10.4; 14.7)

7.7 (5.8; 9.6)

11.7 (9.2; 14.2)

9.3 (6.8; 11.7)

23.0 (16.2; 29.9)

41

0.88 (0.81; 0.96)

0.80 (0.71; 0.90)

21.8

0.70 (0.59; 0.84)

0.69 (0.56; 0.86)

45-49

2.5 (1.5,3.5)

58.4 ± 84.0

13.5 (9.5; 17.4)

10.5 (7.0; 14.0)

16.3 (11.9; 20.7)

12.7 (8.4; 17.1)

33.7 (20.2; 47.1)

39.5

0.83 (0.72; 0.97)

0.65 (0.52; 0.81)

28.1

0.50 (0.34; 0.73)

0.42 (0.27; 0.68)

Rural

2.5 (1.5,3.5)

76.7 ± 89.4

*reference*

*reference*

*reference*

*reference*

*reference*

37.8

*reference*

*reference*

19.7

*reference*

*reference*

Urban

2.5 (1.5,3.5)

86.4 ± 87.9

4.0 (3.0; 4.9)

-0.5 (-1.3; 0.3)

-0.3 (-1.3; 0.8)

-0.5 (-1.5; 0.6)

0.6 (-2.4; 3.6)

28.5

0.87 (0.84; 0.91)

0.98 (0.93; 1.02)

19.4

1.09 (1.02; 1.18)

1.02 (0.93; 1.12)

Poorest

1.5 (1.5,3.5)

64.7 ± 81.3

*reference*

*reference*

*reference*

*reference*

*Reference*

40.1

*reference*

*reference*

29.6

*reference*

*reference*

Poorer

2.5 (1.5,3.5)

69.2 ± 85.2

0.5 (-1.1; 2.0)

-2.5 (-3.9; -1.2)

-2.3 (-3.8; -0.8)

-1.1 (-2.5; -0.4)

-2.6 (-7.6; 2.3)

38.8

0.99 (0.93; 1.05)

0.99 (0.92; 1.06)

25.7

1.08 (0.95; 1.24)

1.08 (0.93; 1.26)

Middle

2.5 (1.5,3.5)

75.3 ± 87.9

0.9 (-0.6; 2.4)

-4.2 (-5.5; -2.8)

-5.3 (-6.8; -3.7)

-2.2 (-3.7; -0.8)

-7.7 (-12.6; -2.8)

37.4

1.02 (0.97; 1.09)

1.07 (0.99; 1.15)

22.5

1.20 (1.05; 1.36)

1.16 (0.99; 1.35)

Richer

2.5 (1.5,3.5)

82.8 ± 90.3

4.2

     (2.7; 5.7)

-4.3 (-5.6; -2.9)

-5.9 (-7.5; -4.2)

-1.9 (-3.5; -0.3)

-10.2 (-15.3; -5.1)

32.8

0.97 (0.91; 1.02)

1.08 (1.00; 1.17)

20.7

1.37 (1.21; 1.55)

1.36 (1.16; 1.59)

Richest

2.5 (1.5,3.5)

93.1 ± 90.1

10.8 (9.3; 12.3)

-3.8 (-5.1; -2.4)

-7.0 (-8.9; -5.2)

-2.9 (-4.7; -1.1)

-16.2 (-21.9; -10.6)

27

0.79 (0.75; 0.84)

1.03 (0.95; 1.13)

15.4

1.37 (1.21; 1.55)

1.37 (1.15; 1.62)

None

1.5 (0.5,3.5)

63.0 ± 83.9

*reference*

*reference*

*reference*

*reference*

*Reference*

44.9

*reference*

*reference*

22.6

*reference*

*reference*

Primary

2.5 (1.5,3.5)

68.1 ± 83.1

6.5 (4.9; 8.0)

4.8 (3.4; 6.2)

4.3 (2.6; 6.0)

1.6 (-0.1; 3.2)

6.9 (1.1; 12.7)

36.4

0.84 (0.79; 0.89)

0.95 (0.88; 1.02)

19.2

0.91 (0.78; 1.06)

0.80 (0.66; 0.96)

Secondary

2.5 (1.5,3.5)

87.9 ± 90.5

10.3 (8.9; 11.8)

3.8 (2.5; 5.1)

4.0 (2.2; 5.7)

1.3 (-0.4; 2.9)

10.4 (4.8; 16.1)

29.3

0.77 (0.73; 0.82)

0.97 (0.90; 1.05)

20

0.95 (0.83; 1.09)

0.72 (0.60; 0.87)

Higher

2.5 (1.5,4.5)

103.4 ± 89.8

18.5 (16.7; 20.4)

2.2 (0.6; 3.9)

1.8 (-0.3; 4.0)

1.4 (-0.8; 3.5)

5.5 (-0.8; 11.9)

23.8

0.62 (0.57; 0.67)

0.88 (0.80; 0.98)

17

1.04 (0.90; 1.21)

0.73 (0.59; 0.90)

Currently married

2.5 (1.5,3.5)

81.5 ± 88.9

*reference*

*reference*

*reference*

*reference*

*Reference*

27.6

*reference*

*reference*

20.6

*reference*

*reference*

Never married

2.5 (1.5,3.5)

73.6 ± 88.7

4.0 (2.0; 6.1)

4.6 (2.8; 6.4)

4.0 (2.0; 6.1)

2.4 (0.4; 4.4)

13.1 (6.2; 19.9)

33.9

0.90 (0.83; 0.98)

0.91 (0.83; 1.01)

19.5

0.96 (0.81; 1.14)

1.00 (0.82; 1.20)

Formerly married

2.5 (1.5,3.5)

76.0 ± 86.5

2.4 (0.6; 4.2)

4.0 (2.4; 5.7)

3.1 (1.3; 4.8)

1.9 (0.2; 3.7)

6.2 (1.3; 11.1)

29.9

0.91 (0.84; 0.97)

0.91 (0.84; 0.99)

21

0.88 (0.77; 1.00)

0.92 (0.80; 1.06)

Female

2.5 (1.5,3.5)

80.4 ± 89.1

*reference*

*reference*

*reference*

*reference*

*Reference*

33.4

*reference*

*reference*

20.1

*refernce*

*refernce*

male

2.5 (1.5,3.5)

82.0 ± 88.6

1.9 (1.0; 2.8)

0.9 (0.2; 1.7)

1.4 (0.6; 2.3)

0.9 (0.1; 1.8)

3.8 (1.3; 6.3)

33.9

1.0 (0.96; 1.03)

1.01 (0.97; 1.06)

19

1.05 (0.98; 1.13)

1.08 (1.00; 1.17)

1

2.5 (1.5,3.5)

92.8 ± 91.7

*reference*

*reference*

*reference*

*reference*

*Reference*

28.2

*reference*

*reference*

17.7

*reference*

*reference*

2-3

2.5 (1.5,3.5)

84.4 ± 89.7

-4.3 (-5.4; -3.3)

-2.1 (-3.1; 1.2)

-3.9 (-5.1; -2.8)

-3.6 (-4.8; -2.4)

-2.6 (-5.7; 0.5)

31.8

1.11 (1.07; 1.17)

1.12 (1.06; 1.19)

19.6

0.92 (0.85; 1.00)

1.01 (0.92; 1.12)

4-6

1.5 (1.0,3.5)

59.1 ± 78.6

-7.4 (-7.8; -6.1)

-0.2 (-1.4; 0.9)

-4.7 (-6.4; -3.0)

-4.7 (-6.3; -3.0)

-1.6 (-6.7; 3.5)

42.7

1.12 (1.13; 1.25)

1.20 (1.11; 1.30)

27.2

0.89 (0.80; 1.00)

1.12 (0.96; 1.31)

7+

1.5 (0.6,3.5)

47.1 ± 69.0

-4.7 (-6.6; -2.7)

1.7 (0.0; 3.5)

-6.6 (-9.2; -3.9)

-5.7 (-8.3; -3.2)

-3.9 (-12.5; 4.7)

48

1.15 (1.07; 1.24)

1.23 (1.09; 1.39)

22.4

0.75 (0.62; 0.91)

1.07 (0.83; 1.40)

Wanted then

2.5 (1.5,3.5)

83.6 ± 89.5

*reference*

*reference*

*reference*

*reference*

*Reference*

32.5

*reference*

*reference*

19.6

*reference*

*reference*

Wanted later

2.5 (1.5,3.5)

75.3 ± 86.6

-1.8 (-3.0; -0.6)

0.7 (-0.4; 1.7)

1.1 (0.0; 2.3)

1.0 (-0.1; 2.1)

-0.3 (-3.8; 3.1)

36.3

1.14 (1.09; 1.19)

1.13 (1.07; 1.19)

17.1

1.03 (0.94; 1.13)

1.04 (0.94; 1.15)

Wanted no more

2.5 (1.5,3.5)

69.4 ± 84.9

-1.5 ( -2.9; -0.1)

2.9 (1.7; 4.2)

2.0 (0.6; 3.5)

0.9 (-0.5; 2.3)

2.2 (-2.2; 6.6)

39

1.06 (1.00; 1.12)

1.03 (0.97; 1.11)

23.5

0.90 (0.80; 1.01)

1.00 (0.88; 1.15)

Not Applicable

**Vaginal births: length of stay <24 hours**

**Cesarean deliveries: length of stay <72 hours**

Not Applicable

Not Applicable

Not Applicable

**All births length of stay in hours**

**Birth order of**

**index child**

**Survival of**

**Index child**

**Women’s age**

**in years**

**Wealth**

**quintile**

**Marital status**

**Sex of child**

**Completed**

**education level**

**Wantedness**

**Birth**

**attendant**

**Multiple birth**

**Birthweight**

**Sector of**

**Facility**

**Residence**
