# Supplementary material for: Length of Stay After Childbirth in 92 Countries and Associated Factors in 30 Low- and Middle-Income Countries: Compilation of Reported Data and a Cross-sectional Analysis from Nationally Representative Surveys
Source: PLoS Med. 2016 Mar 8;13(3):e1001972. doi: 10.1371/journal.pmed.1001972 (PMC4783077; doi:10.1371/journal.pmed.1001972)
Supplement: S3 Table — (DOCX) [file pmed.1001972.s010.docx]

| **S3 Table. Linear regression models for sensitivity analyses. Model A: multivariable linear regression when birthweight is excluded (accounting for most missing data in the main analysis), adjusted for country and all other covariates. Model B: association between factors and log transformed length of stay, adjusted for country and all other covariates. Regression coefficients represent the ratio of the geometric means between each category and baseline.** | | | | | |
| --- | --- | --- | --- | --- | --- |
| **FACTOR** | **Categories** | **Sensitivity Model A** | **P value** | **Sensitivity Model B** | **P value** |
| **Need-related characteristics** | | | | | |
| **Mode-of-delivery** | Vaginal | *reference* | <0.001 | *reference* | <0.001 |
|  | Cesarean | 84.8 (83.7; 85.9) |  | 2.88 (2.82; 2.93) |  |
| **Multiple birth** | Singleton | *reference* | <0.001 | *reference* | <0.001 |
|  | Twins or Triplets (2+) | 27.9 (24.9; 40.0) |  | 1.34 (1.26; 1.41) |  |
| **Birthweight** | <1,999 grams |  |  | 1.25 (1.20; 1.31) | <0.001 |
|  | 2,000-2,499 grams |  |  | 1.09 (1.06; 1.11) |  |
|  | 2,500+ grams |  |  | *Reference* |  |
| **Survival**  (index child) | Died before/on day of discharge | 28.6 (24.9; 32.4) | <0.001 | 1.39 (1.27; 1.52) | <0.001 |
|  | Survived | *Reference* |  | *Reference* |  |
|  | Died after discharge | 1.3 (-1.4; 4.0) |  | 1.00 (0.94; 1.05) |  |
| **Facility/provider characteristics** | | | | | |
| **Birth-attendant** | Nurse-midwife | *Reference* | <0.001 | *Reference* | <0.001 |
|  | Doctor | 21.3 (20.0; 22.5) |  | 1.31 (1.28; 1.34) |  |
|  | Auxiliary staff/other | -2.9 (-5.6; -0.2) |  | 0.90 (0.85.; 0.96) |  |
| **Sector of Facility** | Public | *Reference* | <0.001 | *Reference* | 0.018 |
|  | Private | -4.8 (-5.8; -3.8) |  | 0.98 (0.96; 1.00) |  |
| **Woman’s characteristics** | | | | | |
| **Woman’s age**  (years) | 15-19 | -0.6 (-2.3; 1.1) | <0.001 | 1.00 (0.97; 1.03) | <0.001 |
|  | 20-24 | *Reference* |  | *Reference* |  |
|  | 25-29 | 2.5 (1.3; 3.6) |  | 1.03 (1.01; 1.05) |  |
|  | 30-34 | 5.0 (3.7; 6.4) |  | 1.07 (1.05; 1.10) |  |
|  | 35-39 | 7.7 (6.1; 9.4) |  | 1.12 (1.09; 1.15) |  |
|  | 40-44 | 11.8 (9.5; 14.0) |  | 1.17 (1.13;1.22) |  |
|  | 45-49 | 15.0 (11.2; 18.8) |  | 1.29 (1.20; 1.38) |  |
| **Residence** | Rural | *Reference* | 0.976 | *Reference* | 0.971 |
|  | Urban | 0.0 (-0.9; 0.9) |  | 1.00 (0.98; 1.02) |  |
| **Wealth quintile** | Poorest | *reference* | <0.001 | *Reference* | <0.001 |
|  | Poorer | -3.0 (-4.4; -1.6) |  | 0.99 (0.97; 1.01) |  |
|  | Middle | -5.2 (-6.6; -3.9) |  | 0.96 (0.93; 0.98) |  |
|  | Richer | -6.1 (-7.5; -4.6) |  | 0.95 (0.93; 0.98) |  |
|  | Richest | -6.5 (-8.2; -4.9) |  | 0.96 (0.93; 0.99) |  |
| **Completed education level** | None | *reference* | <0.001 | *reference* | <0.001 |
|  | Primary | 5.4 (4.0; 6.8) |  | 1.06 (1.04; 1.09) |  |
|  | Secondary | 5.1 (3.7; 6.5) |  | 1.06 (1.03; 1.08) |  |
|  | Higher | 2.8 (0.9; 4.7) |  | 1.08 (1.04; 1.11) |  |
| **Marital status** | Currently-married | *reference* | <0.001 | *Reference* | <0.001 |
|  | Never-married | 4.4 (2.5; 6.3) |  | 1.05 (1.02; 1.09) |  |
|  | Formerly-married | 3.1 (1.5; 4.8) |  | 1.04 (1.01; 1.07) |  |
| **Child-related characteristics** | | | | | |
| **Sex of child** | Female | *reference* | 0.018 | *Reference* | <0.001 |
|  | Male | -0.9 (-1.7; -0.2) |  | 0.98 (0.97; 0.99) |  |
| **Birth order (index child)** | 1 | *reference* | <0.001 | *Reference* | <0.001 |
|  | 2-3 | -3.9 (-4.9; -2.8) |  | 0.94 (0.92; 0.95) |  |
|  | 4-6 | -5.4 (-6.9; -3.9) |  | 0.90 (0.88; 0.93) |  |
|  | 7+ | -7.6 (-9.9; -5.3) |  | 0.88 (0.85; 0.92) |  |
| **Wantedness** | Wanted then | *reference* | <0.001 | *Reference* | 0.032 |
|  | Wanted later | 1.3 (0.2; 2.4) |  | 0.99 (0.97; 1.00) |  |
|  | Wanted no more | 2.3 (1.0; 3.6) |  |  |  |
