# Supplementary material for: Length of Stay After Childbirth in 92 Countries and Associated Factors in 30 Low- and Middle-Income Countries: Compilation of Reported Data and a Cross-sectional Analysis from Nationally Representative Surveys
Source: PLoS Med. 2016 Mar 8;13(3):e1001972. doi: 10.1371/journal.pmed.1001972 (PMC4783077; doi:10.1371/journal.pmed.1001972)
Supplement: S1 Text — (DOCX) [file pmed.1001972.s011.docx]

**S1 Text. Categorisation of factors**

All variables used in the analyses were based on women’s reports unless otherwise specified.

**Survival of the index child relative to discharge**

Newborns who subsequently died had their ages at death recorded in days if less than one month, in months up to two years, and in years subsequently. We were interested in understanding the length of stay for women whose newborns who survived, who died up to the time of discharge or who died after discharge. The gradations of age at death differed from those used for length of stay. In our analyses children who died on day zero were considered to have died on the day of discharge for women who stayed <24 hours, and before the day of discharge of all other women. Deaths on day one were determined to have occurred after the discharge for women who stayed <24 hours, but on or before the day of discharge for women who stayed one day or more, etc., up to deaths on day 6. Deaths on days 7-13 were considered to have occurred before or on the day of discharge of women who reported a length of stay of one week, up to day 21-27 of death, which coincided with a three-week length of stay, after which we truncated our dataset. Depending on the length of stay at discharge and the age at death, woman were coded as having a child that survived until the survey, died on/before discharge, or died subsequently.

**Birthweight of the index child** was copied from the child’s health card where available, or was recalled by the mother.

**Birth-attendant** (nurse-midwife, doctor, other auxiliary staff, husband/ friend/ other, no one) and **sector** (public, private) were grouped as detailed previously [^37^](#_ENREF_37).

**Wealth quintile** was based on the DHS’s quintile of asset ownership in women’s households, an approach commonly used to classify socioeconomic position.[^38-40^](#_ENREF_38).

**Sex of index child, birth order and survival of the child for multiple births**. For women with multiple births (twins or triplets), the sex, birth order, and survival of the child was taken for the first multiple reported, and for which interviewers were given no specific instruction. In half of dizygotic twins, each child could have a different sex.

**Wantedness of the index child** refers to whether the pregnancy resulting in the index child was wanted at the time it occurred, was wanted but mistimed, or was not wanted at all.
